# Population expansion, divergence, and persistence in Western Fence Lizards (*Sceloporus occidentalis*) at the northern extreme of their distributional range

**DOI:** 10.1038/s41598-022-10233-9

**Published:** 2022-04-15

**Authors:** Hayden R. Davis, Simone Des Roches, Roger A. Anderson, Adam D. Leaché

**Affiliations:** 1 Department of Biology and Burke Museum of Natural History and Culture, University of Washington, Seattle, WA 98195 USA; 2 School of Aquatic and Fisheries Sciences, University of Washington, Seattle, WA 98195 USA; 3 Department of Biology, Western Washington University, Bellingham, WA 98225 USA

**Keywords:** Evolutionary genetics, Molecular evolution, Phylogenetics, Population genetics

## Abstract

Population dynamics within species at the edge of their distributional range, including the formation of genetic structure during range expansion, are difficult to study when they have had limited time to evolve. Western Fence Lizards (*Sceloporus occidentalis*) have a patchy distribution at the northern edge of their range around the Puget Sound, Washington, where they almost exclusively occur on imperiled coastal habitats. The entire region was covered by Pleistocene glaciation as recently as 16,000 years ago, suggesting that populations must have colonized these habitats relatively recently. We tested for population differentiation across this landscape using genome-wide SNPs and morphological data. A time-calibrated species tree supports the hypothesis of a post-glacial establishment and subsequent population expansion into the region. Despite a strong signal for fine-scale population genetic structure across the Puget Sound with as many as 8–10 distinct subpopulations supported by the SNP data, there is minimal evidence for morphological differentiation at this same spatiotemporal scale. Historical demographic analyses suggest that populations expanded and diverged across the region as the Cordilleran Ice Sheet receded. Population isolation, lack of dispersal corridors, and strict habitat requirements are the key drivers of population divergence in this system. These same factors may prove detrimental to the future persistence of populations as they cope with increasing shoreline development associated with urbanization.

## Introduction

Population divergence occurs across a broad range of spatial and temporal scales. The expected pattern of genetic variation, however, depends partly on whether the populations are located at the periphery or core of the range^1,2^. As a consequence of their smaller size, peripheral populations often have lower genetic diversity compared to core populations^3,4^. Furthermore, the physiological constraints experienced by peripheral lineages can limit dispersal and gene flow among and between populations, which can restrict them to smaller geographic areas. As a result, peripheral populations can be relatively homogeneous with low genetic diversity due to low levels of dispersal and gene flow. Diversification at microgeographic scales is often correlated with either environmental heterogeneity or minor phenotypic changes (e.g., in life history) that contribute to a reduction in gene flow among populations^5–8^. Diversification at fine spatial scales can also occur when a species occupies a non-optimal niche or reaches the edge of a geographic range causing populations to become geographically fragmented, resulting in population structure^9,10^.

Intraspecific diversity is also a product of ongoing and historic environmental changes, including climate driven habitat change which can alter the genetic composition of a population^11^. Populations near the edges or at the extremes of their preferred habitats and environments run up against physiological limitations or stressors, such as thermal maxima or minima, that can cause allele frequencies to shift as populations become smaller and more fragmented^3,12,13^. The low genetic diversity of species already at their physiological limits further restricts adaptive potential in the face of abiotic stressors^14,15^. However, this central-peripheral hypothesis is not always supported in natural systems^3,16^, and there is even evidence of groups excelling at the periphery^17^. Founder events and novel abiotic pressures can work together to shape patterns of genetic variation and population structure observed within species at the edge of their range.

**Figure 1:**
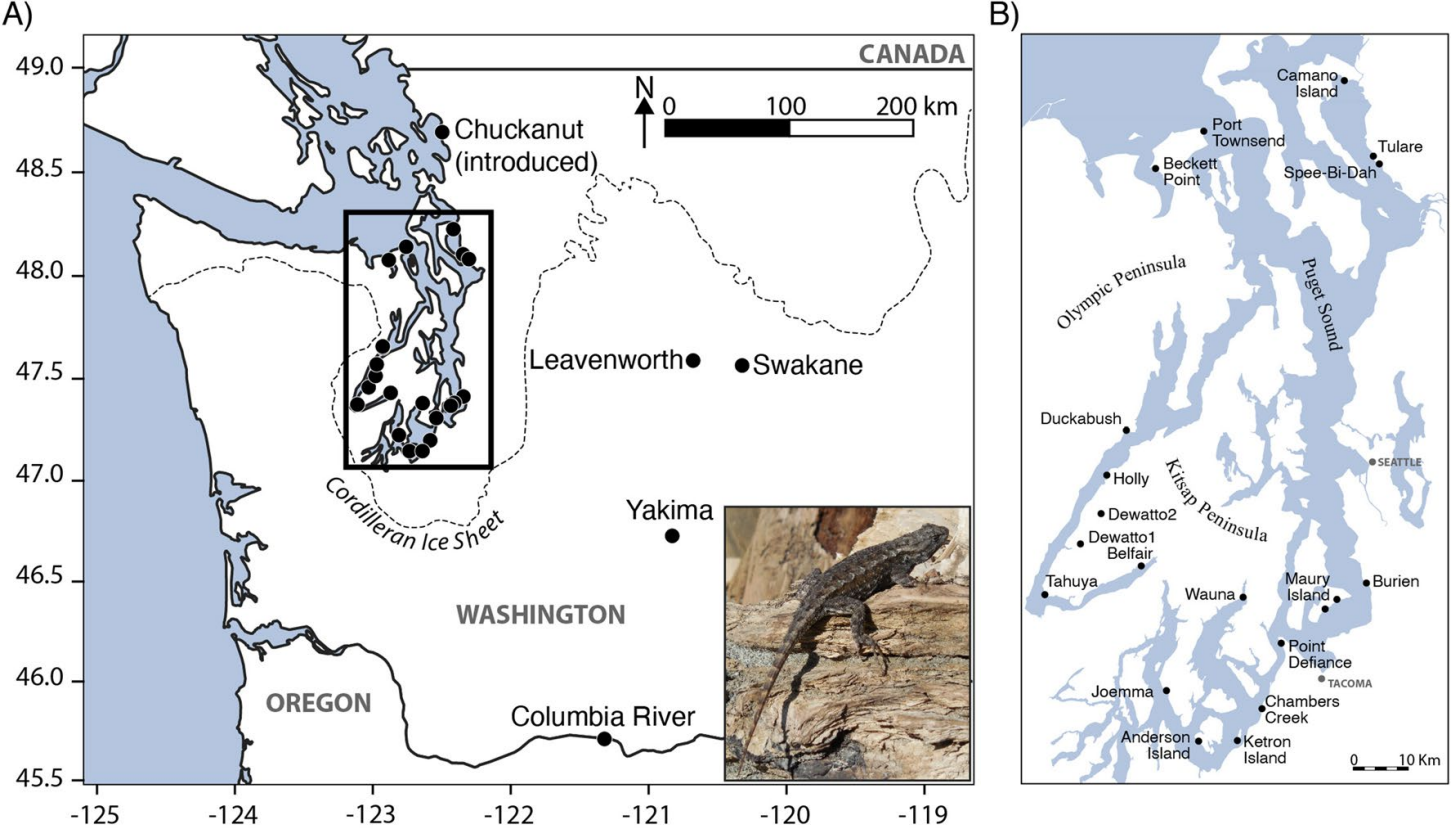
Map of study area. (**A**) *Sceloporus occidentalis* sampled in Washington with the dashed line indicating the maximum extent of the Cordilleran Ice Sheet (16 kya)^76^. Inset figure shows an individual of *S. occidentalis* on driftwood along the coastline of Tulare Beach. (**B**) Detailed sampling map of the Puget Sound region. Sampled locations are labeled with black dots. Map generated using QGIS^77^.

The Western Fence Lizard (*Sceloporus occidentalis*) spans a large geographic area in the Western North America, extending from Baja California, Mexico into the Puget Sound region, Washington^18,19^. Across this relatively large distribution, at least five distinct genetic groups can be identified that are separated by major biogeographic barriers^18^. The Pacific Northwest (PNW) group comprises the northernmost populations, extending from Northern California into Oregon and Washington, and it shares a most recent common ancestor with populations from the Western Sierra Nevadas^18,20^. Throughout much of their central and southern range, populations of *S. occidentalis* are geographically continuous and successful in nearly all habitats, exploiting both urban and natural environments. Yet, populations in Washington have a fragmented distribution, occurring solely along the Columbia River near the Oregon border, in the Cascade Mountains in central Washington, and on shoreline habitats around the Puget Sound in Western Washington (Fig. 1).

Nowhere is the fragmented distribution of *S. occidentalis* more pronounced than at the northernmost extent of the PNW group around the Puget Sound where the species is restricted to small, isolated localities scattered across islands and coastal habitats (Fig. 1, Figs.  S1, S2). With current temperatures in western Washington being lower than temperatures farther south in the species range, reduced fitness in the midst of a colder climate may be restricting their northern distribution^21–23^, as well as limiting the number of suitable habitats. Past climatic events have also presumably played a large role in shaping the distribution of the species, particularly the expansion of the Cordilleran Ice Sheet, which covered the entirety of the Puget Sound as recently as 16 kya^24,25^. As a result, the colonization, or recolonization, of the Puget Sound by *S. occidentalis* has presumably been restricted until the Holocene glacial retreat. Therefore, *S. occidentalis* distributed around the Puget Sound may lack genetic structure due to a combination of factors including recent colonization, range edge effects, and small population size.

In contrast to California populations, few studies have focused on the ecology and life history of *S. occidentalis* at the northern edge of their range. The species is diurnal and a general insectivore with little indication of dietary specialization^26^. However, the Washington populations differ substantially in many aspects of their life history. Washington populations have been documented hibernating between approximately September and May, leading to an increased standard metabolic rate during their active months^27^. The populations in Washington also have increased clutch sizes in comparison to California (12 eggs/clutch vs. 7 in California) that tend to hatch more quickly, presumably due to their smaller size^22^. Despite physiological comparisons of northern to central or southern populations, few studies have directly addressed the ecological or habitat requirements for the northern populations^28–30^. In addition to investigating the genomic and morphological variation of *S. occidentalis* around the Puget Sound, we also aim to provide new information regarding their microhabitat requirements in this region.

We present the first comprehensive study of the distribution of *S. occidentalis* in the Puget Sound and use genome wide single nucleotide polymorphisms (SNPs) and morphological data to test whether population divergence is present in the region. We then describe patterns of genetic and morphological variation and use demographic models to understand how populations have evolved through time. Lastly, since *S. occidentalis* in the Puget Sound region is less abundant and more fragmented than other populations throughout the species distribution, we discuss the ecological requirements necessary for their success at the northernmost extent of their range. These new data provide strong support for multiple genetically distinct groups in the Puget Sound region, which has important implications for conservation and population management.

## Methods

### Ethics statement

The research presented herein was conducted in accordance with the ethical standards and guidelines outlined by the University of Washington’s Institutional Animal Care and Use Committee (IACUC protocol #4367-02). Samples were collected with permission from the Washington Department of Fish and Wildlife (WDFW permit 20-144).

### Study system and taxon sampling

We collected *Sceloporus occidentalis* individuals from 19 localities across the Puget Sound Region and four localities from surrounding regions in Eastern Washington, with most sampling being conducted in 2020 (Fig. 1; Table 1). We captured lizards using a three meter fishing rod with a thin loop on the end, which was placed over the head of the lizard and subsequently tightened. In total, we incorporated 90 samples in our study with 78 of those from the Puget Sound region (Table 1). Voucher specimens and tissue samples are accessioned at the Burke Museum of Natural History and Culture (UWBM:HERP:10034–10111; Table S1). Our morphological and genomic datasets are mostly overlapping with a few exceptions: when the specimen wasn’t collected or was a juvenile, we did not collect morphology data; when we had low sequencing coverage for an individual, we removed it from the genetic dataset.

**Table 1:** Sample locations and sample sizes. Detailed voucher specimen information is provided in Table S1.

Location	Samples
**Puget Sound Region**	
Anderson Island	4
Beckett	4
Belfair	2
Burien	3
Camano Island	4
Cambers Creek	6
Chuckanut	4
Dewatto	6
Duckabush	4
Holly	4
Joemma	4
Ketron Island	4
Maury Island	5
Point Defiance	3
Port Townsend	4
SpeeBiDah	2
Tahuya	4
Tulare	7
Wauna	4
**Eastern Washington**	
Columbia River	4
Leavenworth	1
Swakane Canyon	5
Yakima	2
**Oregon**	
Skunk Hollow	2
Selma	2
Shaniko	2

### Molecular methods

We extracted genomic DNA from liver biopsies using salt-extraction^31^ and then conducted double digest restriction-site associated DNA sequencing (ddRADseq)^32^. We double-digested each sample using the digestion enzymes SbfI and MspI in CutSmart Buffer (New England Biolabs) for 7 h at 37 $^\circ$C. For fragment purification, we used Sera-Mag SpeedBeads. We then prepared a master mix for eight distinct barcodes to be ligated to the cut sites of the fragmented DNA. The libraries were size-selected (between 415 and 515 bp after accounting for adapter length) on a Blue Pippin Prep size fractionator (Sage Science). For the final library amplification, we used Phusion Hi-Fidelity DNA Polymerase and Illumina’s indexed primers. We determined the concentration and size distribution of each indexed pool using an Agilent 2200 TapeStation. Lastly, we sent the quantified pools to QB3-Berkeley Genomics, UC Berkeley for qPCR to determine sequenceable library concentrations before multiplexing equimolar amounts of each pool for sequencing on one Illumina HiSeq 4000 lane (51-bp, single-end reads; 11 pools containing up to 8 samples each). The demultiplexed sequences are deposited at the Sequence Read Archive (NCBI-SRA; BioProject ID: PRJNA757434; Table S1).

### Bioinformatics

We demultiplexed each sample from their respective pool using their unique barcode sequence using iPyRAD v.0.9.50^33^. We conducted a reference-based assembly of the RAD loci using a draft of the *S. occidentalis* genome from Yosemite National Park, California^34,35^ (Table 2). A sequence similarity threshold of 90% was used to cluster reads within samples and loci between samples. We removed consensus sequences with low coverage (< 6 reads), excessive undetermined or heterozygous sites (> 5%), too many alleles for a sample (> 2 for diploids), or an excess of shared heterozygosity among samples (paralog filter = 0.5). For the final alignments we generated output files containing 0% missing data (1037 loci) and 50% missing data (3491 loci; Table 2, Table S2). Downstream population genetic analyses used additional filtering to subsample one random SNP per locus, and those datasets are described below.

**Table 2:** The number of SNPs obtained from the reference-based assembly of 78 samples of *Sceloporus occidentalis* from the Puget Sound Region. Allowing for more missing data (50%) results in more SNPs compared to no missing data (0%).

Chromosome	50% missing data	0% missing data
chr1	322	62
chr2	291	60
chr3	218	37
chr4	223	48
chr5	143	22
chr6	170	35
chr7	64	15
chr8	51	13
chr9	47	12
chr10	8	2
chr11	17	3
Total SNPs	1554	309

### Genomic differentiation

We conducted genetic clustering analyses to estimate population structure. Using the 50% missing dataset, we ran a principal component analysis (PCA) using the R package ‘adegenet’ to establish general patterns of genetic diversity, and a discriminant analysis of principal components (DAPC) to determine if a sample’s locality could be determined by genomic data^36,37^. We used the program ADMIXTURE^38^ to visualize ancestry within and among populations. This analysis used a reduced dataset containing one randomly sampled SNP from each locus. Due to the shallow divergence and microgeographic scale of the study system, estimating an optimal *K*-value proved difficult. We repeated the ADMIXTURE analysis 10 times for each *K*-value ranging from 1–10 and used the program’s cross-validation (CV) procedure to test for the optimal number of subpopulations (lowest CV = optimal *K*-value). To visualize and compare results, we used the program CLUMPAK^39^ to produce structure barplots. We repeated these procedures for datasets with 0% (236 SNPs) and 50% (707 SNPs total) missing data.

Using the genetic subpopulations identified by the population structure analyses, we quantified the extent of genetic differentiation ($F_{ST}$) between each subpopulation using the option --weir-fst-pop in the program VCFtools^40^. We used the 50% missing data dataset to maximize the number of loci in the analysis. Because the Puget Sound population is not geographically continuous throughout the region, our sampling was often confined to small, restricted geographic areas. To avoid including samples from siblings and other close familial relationships, we calculated the inbreeding coefficient among individuals using the option --relatedness2 in VCFtools^41^. Lastly, we used the program MEGA version X^42^ to calculate nucleotide diversity ($\pi$) among subpopulations.

### Phylogenetic analyses

To estimate the phylogenetic relationships among samples and the timing of population divergence we used a combination of network, concatenated, and coalescent-based phylogenetic approaches. For the network analysis, we used the concatenated SNP data from the 50% missing dataset in the program SplitsTree v. 4.16.1^43^ with the Neighbor-Net algorithm^44^. To explore relationships between individuals and to test for monophyly among subpopulations, we concatenated the RAD loci and constructed a phylogeny using RAxML v8.2.10. For this, we expanded our 50% missing data dataset to include a broader representation of the PNW clade with samples from the Cascade Mountains, Columbia River, and Oregon (Table 1, Table S3). We used a GTR+GAMMA substitution model with 100 rapid bootstraps^45^. Using the same expanded dataset but only including biallelic SNPs, we aimed to estimate divergence times using the multispecies coalescent model in the program SNAPP v1.5.0^46^, implemented in BEAST v2.5.2^47^. To estimate the timing of diversification into the Puget Sound region, we calibrated the species tree using a secondary calibration for the age of the PNW clade of 100 kya^18^. We assigned a prior to calibrate the root of the species tree with a normal distribution, a mean = 100 kya, and a 95% confidence interval of ± 4 kya to accommodate estimation error. This analysis included three additional samples from Oregon, which were downloaded from the NCBI-SRA. We modified the input files for divergence dating in SNAPP using the snapp_prep scripts^48^. To decrease the computation time, we reduced the number of samples from the Puget Sound region to 11, each from unique localities (Table S3). We ran two separate analyses for 200,000 generations each (sampling every 50 generations) to check for convergence across independent runs. We then combined posterior distributions using LogCombiner, and produced a maximum clade credibility (MCC) tree using TreeAnnotator after discarding the first 10% of samples as burn-in.

### Demographic analyses

To investigate population demographics, we analyzed samples from the Puget Sound region as a single population. We performed demographic analyses using the program *Moments*^49^ using one SNP sampled randomly from each locus (707 SNPs total) from the 50% missing data dataset. To maximize the number of segregating sites, we projected the data down to a smaller sample size (*N* = 70) using the program easySFS (https://github.com/isaacovercast/easySFS). We optimized four single-population demographic models using Python scripts^50^: (1) two-epoch model with instantaneous size change (two parameters): $N_{\mu }$ = ratio of contemporary to ancient population size and *T* = time in the past at which size change happened; (2) exponential growth model (two parameters): $N_{\mu }$ and *T*; (3) bottlegrowth with instantaneous size change followed by exponential growth (three parameters): $N_{\mu }$, *T*, and $N_{\mu} {B}$ = ratio of population size after first change to ancient population size; (4) three epoch model with multiple population size changes (four parameters): $N_{\mu }$, $N_{\mu } {B}$, *T*, and *TB* = duration of bottleneck. We performed four rounds of model optimization under each model with 50 replicates each and 25 maximum iterations. For each model, we used the parameters from the best-scoring replicate as starting values for the next round of optimization. After the final optimization, we used the replicate with the highest likelihood for each model to calculate AIC scores and perform model selection^51^. For the top-ranked model, we conducted 100 replicate simulations to assess the goodness-of-fit of the model to the data. We tested the top-ranked model by comparing the empirical log-likelihood value to the values obtained from 100 parametric bootstrap replicates, with the expectation that the empirical value will fall within the range of simulated values. Finally, we obtained confidence intervals for parameters using bootstrapping (100 replicates) by re-sampling the SNP data with replacement, and then optimizing model parameters for each replicate using the same procedure described above. We converted the unscaled population parameters to demographic terms as follows: the time parameter *T* used the equation $\textit{T} = 2 \times N_{ref} \times$ generation time. We used a generation time of 2 years, as observed for the species^52^. To calculate $N_{ref}$, we used the equation $\theta$/4$\mu$*L*, where $\mu$ is the mutation rate and L is the number of loci multiplied by their length. $\theta$ was derived from the *Moments* output and we used a generalized lizard $\mu$ of 7.7e−10^53^.

### Morphological variation

To determine whether there are any patterns of morphological divergence in Puget Sound *S. occidentalis*, we collected morphometric and meristic data from adult individuals. We included most samples used in the genetic dataset, with additional samples from some localities (N = 80; Table S4). We included morphological traits which have been shown to be highly variable in *Sceloporus*, both across species and within populations^54,55^. In total, we collected 15 morphological characters with six morphometric measurements: snout-vent length (SVL), measured from the tip of the snout to the vent; tail length (TL), measured from vent to the end of the tail; head length (HL), measured from the parietal eye to the tip of the rostrum; head width (HW), measured at the widest part of the head; right and left longest toe length (RLL and LLL, respectively), measured from the base to the tip of the toe; right and left femur length (RFL and LFL, respectively), measured from the ventral midline to the distal part of the knee, and five meristic counts: left femoral pores (LFP); right femoral pores (RFP); left longest toe lamellae (LTL); right longest toe lamellae (RTL); medial scales (MS); and dorsal scales (DS).

To determine whether subpopulations within the Puget Sound show any morphometric variation, we performed multivariate statistics using a principal component analysis (PCA) and a linear discriminant analysis (LDA). We determined whether male and female individuals were significantly different by conducting a Mann–Whitney *U* test under the null assumption that the morphological distribution between the two groups are the same. The difference between males and females was statistically insignificant (p = 0.17), thus we analysed all individuals together. We made natural log transformations of all morphometric data. To account for allometric growth, we used R to size-correct the morphometric data by regressing each morphometric trait against SVL for all individuals and using the residuals in subsequent analyses (PCA and LDA)^56,57^. We normalized but did not size correct the discrete meristic data. We analyzed the normalized meristic and size-corrected and log-transformed morphometric data both separately and combined.

### Natural history

From April 1999 through October 2018, ecological data was collected for 466 *Sceloporus occidentalis* individuals in the Puget Sound region. We collected the following data using visual encounter surveys: lizard body temperature, air temperature, lighting conditions, and the microhabitat and substratum that the lizard was observed on. We collected lizard body temperature data using a cloacal thermometer and air temperature using various external thermometers. All data collected were combined with other ecological studies on the population^28,30^. We did not collect ecological data for the lizards used in the genetic study.

## Results

### Geographic distribution

The geographic distribution of *Sceloporus occidentalis* is discontinuous throughout the Puget Sound region. The majority of sites sampled had subpopulations confined to small, geographically isolated stretches of habitat. All but two localities (Dewatto1 and Dewatto2; Fig. 1) from which we found lizard assemblages were on coastlines, with most being south facing, presumably to maximize daily heat and sun exposure. The two Dewatto assemblages were found at deforested inland plots with high sun exposure (Figs. S1 and S2). The northernmost locality sampled is Chuckanut, WA; however, records indicate that this population is not naturally occurring and was transplanted from the Camano Island area^58^. Therefore, the northernmost naturally occurring locality is on Camano Island, although others may have been overlooked. In total, we detected 22 unique localities throughout the Puget Sound region (Fig. 1).

### Genomic differentiation

**Figure 2:**
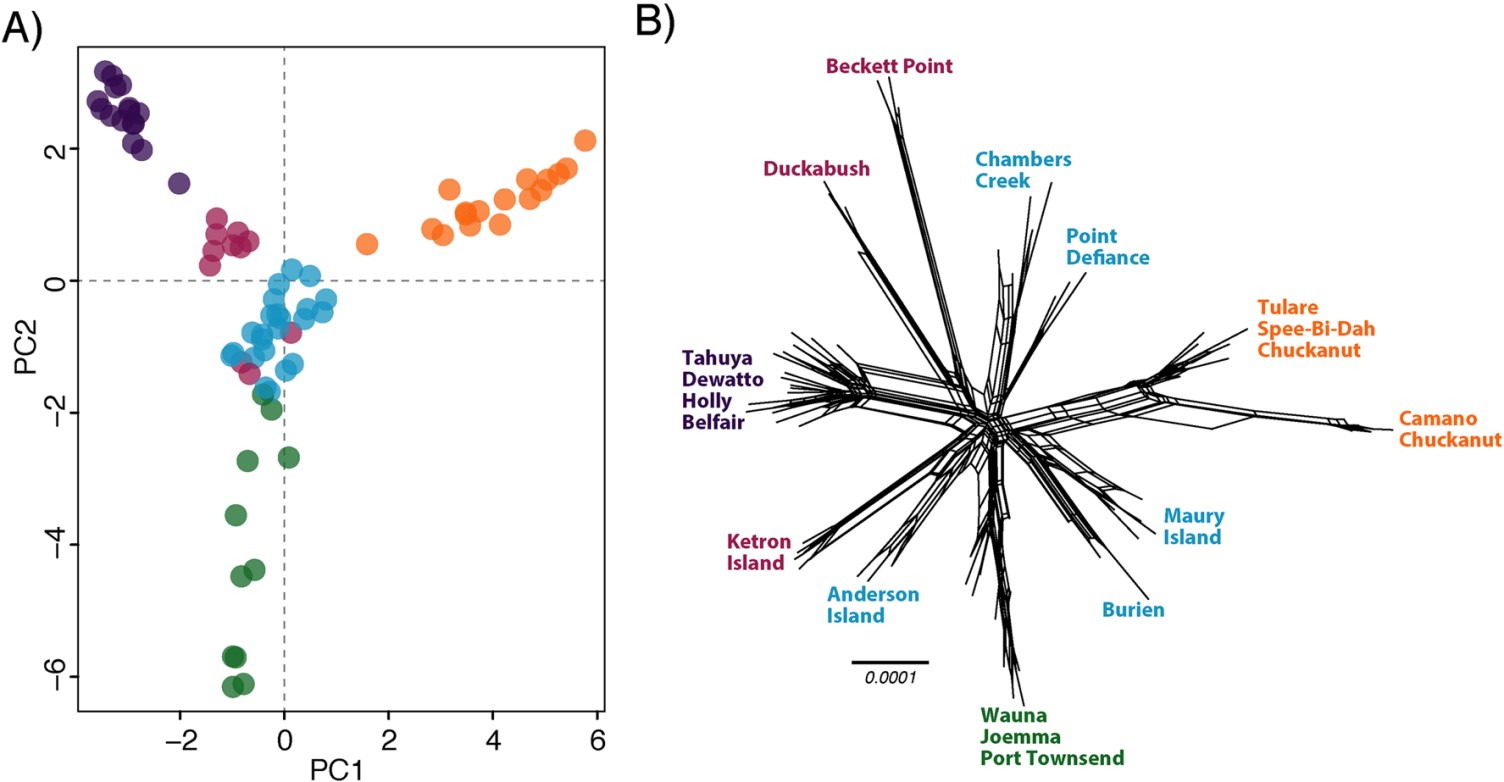
Genetic diversity of *Sceloporus occidentalis* in Western Washington. (**A**) Principal components analysis (PCA) of genetic variation using 3491 loci in the R package ‘adegenet’. (**B**) Network analysis using the same dataset as the PCA demonstrates that samples from the same locality form distinct genetic clusters. Colors correspond to the population assignments from ADMIXTURE assuming *K*-value = 5.

**Figure 3:**
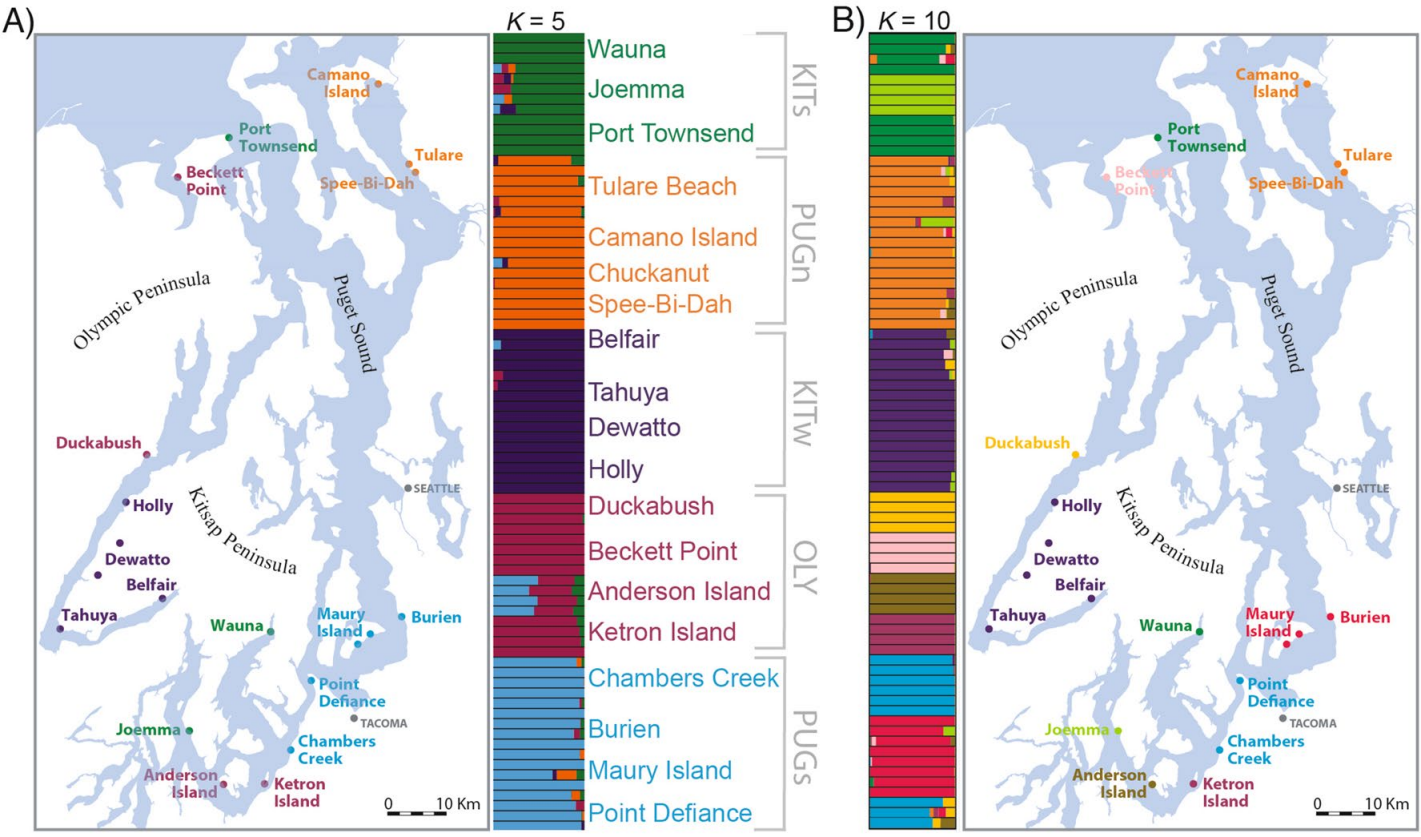
Geographic distribution of populations based on ADMIXTURE analyses assuming a *K*-value = 5 (**A**) and 10 (**B**). Each bar represents an individual, and the colors indicate the admixture proportions. Results are shown for the 50% missing data dataset (3491 loci). Subpopulation names are assigned using a *K*-value = 5. *KITs* Kitsap Peninsula south, *PUGn* Puget Sound north, *KITw* Kitsap Peninsula west, *OLY* Olympic Peninsula, and *PUGs* Puget Sound south. Maps generated using QGIS^77^.

Population structure analyses support multiple genetic clusters within the Puget Sound. For clarity in the following sections, we will refer to five subpopulations found in the Puget Sound region based primarily on the genomic data, while also considering their geographic distributions: Olympic Peninsula (OLY: Duckabush, Beckett Point, Anderson Island, Ketron Island), Puget Sound north (PUGn: Tulare Beach, Camano Island, Chuckanut, Spee-Bi-Dah), Puget Sound south (PUGs: Chambers Creek, Burien, Maury Island, Point Defiance), Kitsap Peninsula west (KITw: Belfair, Tahuya, Dewatto, Holly), and Kitsap Peninsula south (KITs). The PCA reveals at least four genetic groups within the Puget Sound region with most samples clustering with those from geographically proximate areas, except for the KITs group which spans the entire Puget Sound region (Fig. 2). The results from the ADMIXTURE analyses largely corroborate the results of the PCA (Fig. 3). The best-supported cross-validation score for the 50% missing data dataset supported a *K*-value of six; the best-supported *K*-value for the 0% missing data dataset was ten (Fig. S3). Despite the analyses not converging on the same “best” *K*-value, we use a *K*-value of five due to the consistency with the PCA results and the geographic pattern demonstrated (Fig. 2). When a *K*-value of four is used, samples from Beckett Point, Duckabush, and Ketron Island cluster with PUGs. When a K-value of five is used, Beckett point, Duckabush, and sometimes Ketron Island and Anderson Island comprise their own group, which is more consistent with the geographic proximity of the samples (Fig. 3). Intriguingly, as the *K*-value increases, samples from nearly all localities form separate clusters, thus enabling the identification of the specific locality many of the samples were taken from (Fig. 3B). In most cases, identifying the specific locality a sample originates from can also be determined from the DAPC using genomic data (Fig. S4). However, the unique localities comprising PUGn and KITw are difficult to distinguish using either ADMIXTURE with a *K*-value $\ge$ 10 or clusters from a DAPC.

Although the genomic data can be used to pinpoint the specific location of origin for most samples, the genetic diversity around the Puget Sound is, as expected, relatively low. The maximum genome-wide pairwise distance between any two genetic clusters is 0.089%, which is for the comparison between samples from opposite ends of the Puget Sound (Camano Island vs. Anderson Island). The minimum pairwise distance between any two genetic clusters is 0.030%, which is for the comparison between samples from Joemma and Chambers Creek (Table 3). Despite nominal pairwise distances, the $F_{ST}$ values between subpopulations are high, for reasons discussed below (see Discussion). The highest $F_{ST}$ value is between the PUGn and KITs groups (0.17), and lowest between the PUGs and OLY groups (0.065; Table 3). Despite the low genetic diversity and isolated regions from which we collected samples, the genomic data show no signals of first degree relatives, but second and third degree relatives are present (Fig. S5).

**Table 3:** $F_{ST}$ values between genetic groups (upper diagonal) and pairwise distances (lower diagonal) for *Sceloporus occidentalis* in the Puget Sound region. The lowest pairwise distance between any two samples from a given population is shown, rather than an average of all samples from a given locality. The five groups comprising the Puget Sound region are: *OLY* Olympic Peninsula, *KITs* Kitsap Peninsula south, *KITw* Kitsap Peninsula west, *PUGn* Puget Sound north, and *PUGs* Puget Sound south.

	OLY	PUGn	KITs	PUGs	KITw
OLY		0.14	0.12	0.065	0.12
PUGn	7.6e−4		0.17	0.099	0.16
KITs	6.2e−4	5.2e−4		0.096	0.17
PUGs	4.7e−4	4.1e−4	3.0e−4		0.092
KITw	6.8e−4	5.5e−4	4.0e−4	3.2e−4	

### Phylogenetic analyses

The phylogenetic analyses support the general patterns of genetic clustering found in the population structure analyses, wherein many samples are clustered according to their collection locality (Fig. S6). For example, the network analysis clusters samples by collection locality in the PUGs and OLY groups (Fig. 2B). The Camano Island samples are unique from the Tulare Beach and Spee-Bi-Dah samples—the latter two occur on the same stretch of beach and may have a continuous distribution. The Chuckanut population, which was established via human translocation, was derived from multiple source populations in the northern Puget Sound (Fig. 2B, Fig. S6). The samples from the KITs and KITw groups are far less structured in the network analyses, a pattern akin to the ADMIXTURE results.

**Figure 4:**
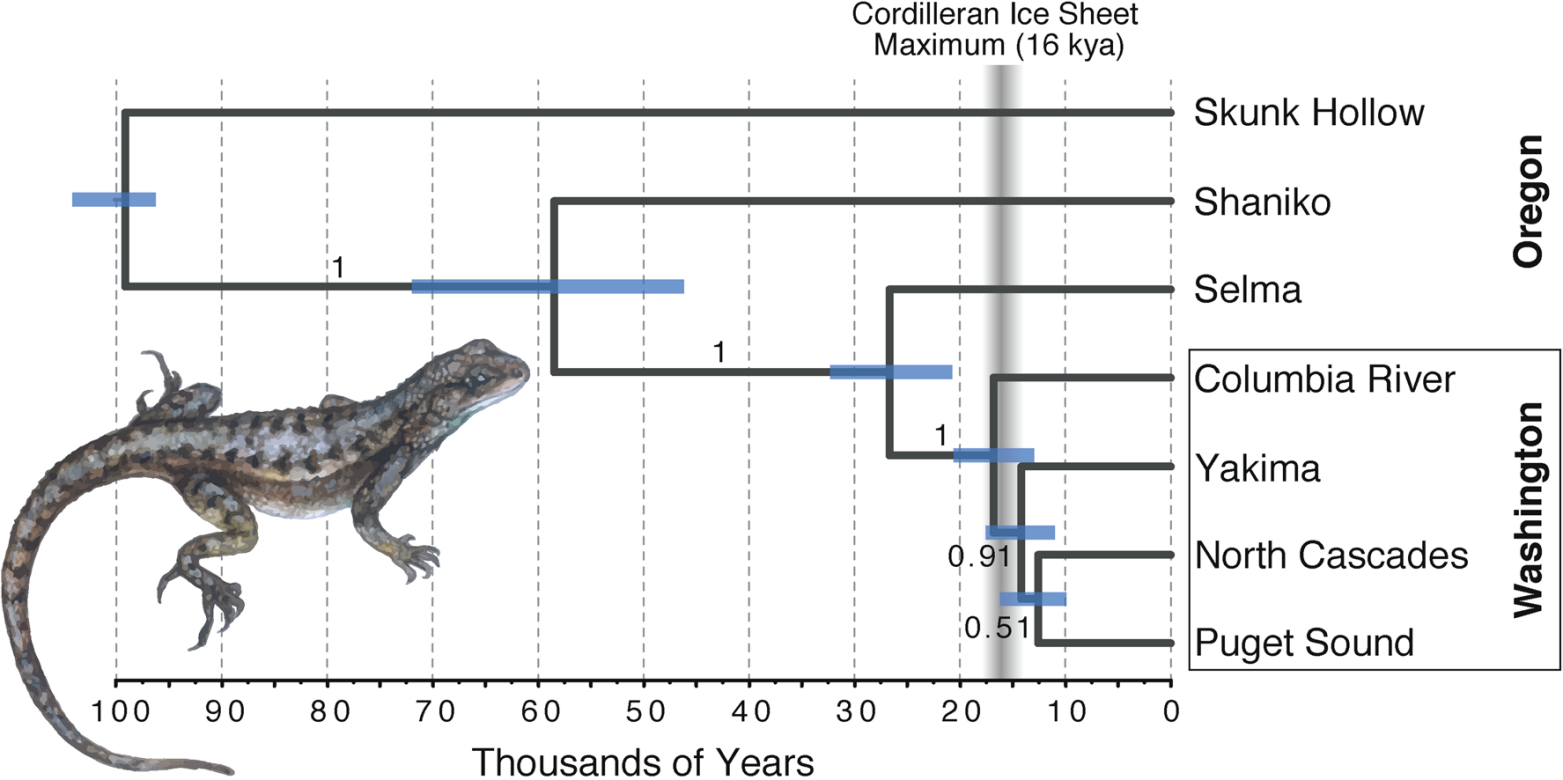
Species tree analysis of *Sceloporus occidentalis* from the Pacific Northwest based on a coalescent analysis of 953 biallelic SNPs. The species tree was calibrated assuming a root divergence time for the Pacific Northwest clade of 100 kya (± 4 kya)^18^. Posterior probability values are shown on branches, and node error bars show 95% highest posterior density (HPD) of divergence times. The timing of deglaciation of the Cordilleran Ice Sheet at 16 kya is shown with a vertical bar.

The species tree topology estimated using SNAPP supports the monophyly of the Washington samples with strong support (posterior probability = 1.0; Fig. 4). The Washington clade diverged from the remainder of the PNW clade 17.21 kya (13.36–20.78 95% HPD), consistent with post-Pleistocene colonization as the Cordilleran Ice Sheet began to recede. The Puget Sound population is weakly supported as sister to the North Cascades population (posterior probability = 0.51), sharing a most recent common ancestor (MRCA) 13.01 kya (10.13–15.96 95% HPD). This clade diverged from the Yakima population 14.50 kya (11.49–17.56 95% HPD), although this relationship is also weakly supported (posterior probability = 0.91). The divergence events in the Washington populations occurred rapidly, as reflected by their relatively short branch lengths (Fig. 4).

### Demographic analyses

Four demographic models were tested (two epoch, growth, bottlegrowth, and three epoch) to understand the population history of *S. occidentalis* in the Puget Sound. The differences in log-likelilhood scores among the optimized models were small (< 0.2 units), and we ranked the models by their AIC scores (Table 4). The top-ranked model was the two epoch model (wAIC = 0.43), followed by the growth (wAIC = 0.36) and bottlegrowth (wAIC = 0.15) models (Table 4; Fig. S7). We present the results of the top two models (two epoch and growth), which account for nearly 80% of the cumulative AIC. Both models infer a recent and substantial population size expansion, but they differ in the magnitude and timing (Table 5). The two epoch model infers a 25.83× population size increase at 10.15 kya (9.10–13.64 95% HPD) and the growth model supports a 16.71× expansion at 16.04 kya (13.95–24.40 95% HDP). The two epoch model indicates that there was a delay between the colonization of the Puget Sound region and the major expansion of the population; whereas the growth model indicates that the colonization and expansion of the population occurred simultaneously.

**Table 4:** Demographic models ranked by AIC scores.

Model	Parameters	Log-likelihood	AIC	$\Delta$AIC	RelativeL	wAIC	CumulativeAIC
Two epoch	2	− 35.38	74.76	0.00	1.00	0.43	0.43
Growth	2	− 35.54	75.08	0.32	0.85	0.36	0.79
Bottlegrowth	3	− 35.43	76.86	2.10	0.35	0.15	0.94
Three epoch	4	− 35.32	78.64	3.88	0.14	0.06	1.00

**Table 5:** The top-ranked demographic models and their optimized model parameter estimates. The 95% confidence intervals were obtained using non-parametric bootstrapping. The time values have been converted into thousands of years using the equation provided in the “Methods” section.

Model	Population size change (95% CI)	Time (95% CI)
Two epoch	25.83× (15.49–46.83)	10.15 (9.10–13.64)
Growth	16.71× (16.35–44.67)	16.04 (13.95–24.40)

### Morphological variation

**Figure 5:**
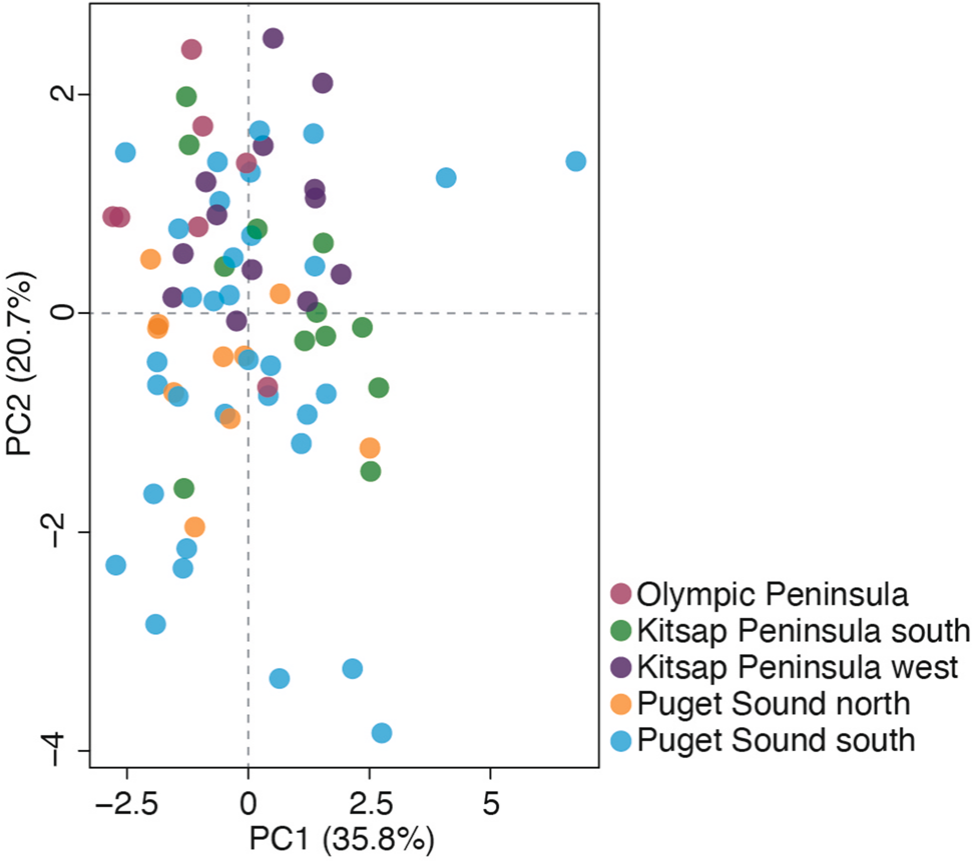
Principal component analysis of morphological data using size-corrected and log-transformed morphometric data and meristic counts for subpopulations from the Puget Sound region in the R package ‘tidyr’. Colors correspond to the population assignments from ADMIXTURE assuming *K*-value = 5.

None of the morphological traits evaluated here can be used to clearly distinguish subpopulations from one another (Table S5). We present results for the combined analyses of the meristic and morphometric data (analyzing these characters separately produces similar results). Principal components one and two account for approximately half of the variation in the dataset (47.4%), with the heaviest loadings on PC1 being the right and left femur lengths, respectively; and the heaviest loading on PC2 being head width (Table S6). Nonetheless, the PCA is unable to distinguish any of the genetic subpopulations using morphometric and meristic data (Fig. 5). Despite lacking clear morphological distinction, the LDA demonstrates that the morphological data can be used to accurately identify which subpopulation a sample originates from with 61.5% accuracy (Table 6), suggesting that a complex combination of characters could be used to identify subpopulations with low accuracy.

**Table 6:** Accuracy of correctly assigning a sample to its genetic group (assuming *K*-value = 5) using morphometric and meristic data in a linear discriminant analysis. The five groups tested from the Puget Sound region are: *OLY* Olympic Peninsula, *KITs* Kitsap Peninsula south, *KITw* Kitsap Peninsula west, *PUGn* Puget Sound north, and *PUGs* Puget Sound south.

	OLY	KITs	PUGs	KITw	PUGn
OLY	4	0	1	1	0
KITs	0	6	2	0	1
PUGs	2	5	26	6	3
KITw	1	1	3	6	0
PUGn	0	0	2	0	6
Accuracy	0.57	0.50	0.76	0.46	0.60

### Natural history

Using data collected from 466 lizards observed between 1999–2020, we provide updated information on the ecology and natural history of *S. occidentalis* in the Puget Sound region. The distribution of the Puget Sound population is primarily restricted to south-facing beaches and occasionally deforested areas that receive high sun exposure. Of the lizards recorded, 78.5% (n = 465) of individuals were in open, sunlit area (no shading or light filtering). On coastlines, we found individuals on driftwood a majority of the time (57.7%; n = 433), with most being on shorelines that meet forested hills. Additionally, despite prior records stating that the populations hibernate from late September through mid May^27^, we observed individuals basking from April through October. These activity windows are presumably dependent on annual climate patterns. Lastly, we observed body temperatures ranging from 19.8–37.8 °C (n = 465), with a mean of 34.1 °C and median of 34.8 °C, consistent with body temperatures documented for the species^59^.

## Discussion

We investigated the geographic distribution, genetic and morphological variation, and phylogenetic and demographic history of *Sceloporus occidentalis* at the northern edge of the species range. The genomic data demonstrate that despite spanning a microgeographic scale and lacking any pronounced ecological differences, the Puget Sound region comprises multiple genetically identifiable subpopulations. Although genetic diversity is relatively low, in many cases there is sufficient genomic differentiation to identify the specific location from which a lizard originated.

Despite low genetic diversity ($\pi$) among subpopulations around the Puget Sound, $F_{ST}$ values are relatively high (Table 3). The high $F_{ST}$ values in the midst of low genomic differentiation is expected due to low heterozygosity in the Puget Sound population as a whole^60^. Considering the low genetic diversity observed in the group, the relatively high $F_{ST}$ values denoted in Table 3 could indicate that minimal gene flow occurs among subpopulations in the Puget Sound region. This conclusion is also supported by the population structure and phylogenetic analyses, which indicate that many of the subpopulations examined are isolated from one another.

The post-glacial colonization of the Puget Sound region by *S. occidentalis* combined with low levels of genetic diversity contribute to a lack of resolution on the optimal number of genomic subpopulations, and difficulty in identifying a consistent *K*-value. There is a large degree of difference between optimal *K*-value inferred for the 50% (*K*-value = 6) and 0% (*K*-value = 10) missing data datasets (Fig. S3). Inferring the true *K*-value for a population is a difficult procedure, and most models are expected to be somewhat inaccurate^61^. We considered population structure models using differing amounts of missing data, and observed that increasing the number of populations in the model typically produced biologically realistic results well beyond any optimal *K*-value. In general, each incremental increase in *K*-value beyond the optimal value provided support for an additional unique sample location as distinct, suggesting that there is sufficient information in the SNP data to distinguish many of the sample locations. Although we present results for a model with *K*-value = 5, we are not definitively stating that five genetic subpopulations occur within the region. Rather, given our genomic dataset, a *K*-value = 5 is a fairly conservative and biologically realistic estimate for the number of subpopulations. Substantially more loci are required to increase the resolution and confidence in the number of subpopulations in the Puget Sound region.

The phylogenetic and population demographic results are consistent with the hypothesis of a Holocene colonization of the Puget Sound region following the retreat of the Cordilleran Ice Sheet. Although the PNW clade split from the Sierra Nevada group 100 kya^18^, the opportunity for *S. occidentalis* to colonize the Puget Sound region was presumably limited until at least 16 kya when the ice sheet began receding. The possibility remains that a population colonized the Puget Sound prior to the expansion of the ice sheet and was subsequently extirpated. Regardless, our results demonstrate that the colonization, or recolonization, of the Puget Sound region occurred approximately 13 kya and subsequently underwent a substantial population expansion (Fig. 4, Table 4). We expect that as the climate continues to warm, more habitat within the Puget Sound region could increase in suitability, thus promoting continued population expansion. Although climate predictions may be favorable for *S. occidentalis* population expansion, the lack of suitable habitats may prohibit further expansions. For example, extensive shoreline development^62^ and urbanization surrounding the Seattle region has likely already limited and fragmented *S. occidentalis* habitats as it has done in the southern part of its range^63^. Further research on the ability of *S. occidentalis* to colonize landscapes in the face of urbanization and shoreline development remains to be studied in this region.

By solely focusing on *S. occidentalis* in the Puget Sound region, we reveal a unique distribution wherein the group spans the majority of the region; however, the distribution comprises many isolated localities as opposed to being continuous. We suspect that the Camano Island locality is the northernmost naturally occurring location for the coastal PNW population. There are species records from farther north and west, but it is unclear whether these records represent introduced and/or extant populations^64^. To our knowledge, *S. occidentalis* does not occur on the northernmost end of the west side of the Olympic Peninsula, as supported by other studies^65^, yet one record exists^66^. If this record represents a natural population, it was likely extirpated. Additionally, in 2020, three records of *S. occidentalis* were made in British Columbia (B.C.), Canada, with two from the citizen-science platform iNaturalist and one from a scientific study^64^. Older records also indicate the presence of *S. occidentalis* in B.C., but with no specific locality information^67^. We expect that these records represent either accidentally or purposefully translocated individuals, yet it remains unclear whether they have or will be able to establish sustainable populations. If more individuals are detected from the region, genomic testing could prove useful in determining their geographic origin.

Temperature can have a drastic effect on physiological function in *S. occidentalis* populations^22^, which may provide an explanation for their fragmented distribution in the Puget Sound region. In addition to females dedicating more resources to rearing offspring at higher latitudes^22^, the northern populations also have substantially reduced physiological growth rates. In a laboratory-based experiment, hatchlings from Deschutes County, Oregon exposed to 34 °C versus 27 °C environments for 12 h had a growth rate 1.4× greater. Given the same conditions but only 6 h of exposure to the specified temperature, the growth rate increased to 1.7× that of the colder temperature^22^. Additionally, Oregon populations showed fewer hours of activity per day than those in California^23^. The opportunity for Puget Sound subpopulations to have extra sunlight provided by inhabiting south facing beaches or full sun areas may be critical for their success. Further, the cool temperatures of the Puget Sound region (average temperature for Seattle in August, the warmest month of the year, is 22 °C), may necessitate exposure to additional hours of warmth for survival. In our field surveys, we only detected two localities (Dewatto1 and Dewatto2, KITw group; Fig. 1) that were not coastal, both of which were in deforested patches with full sun exposure for all hours of daylight. However, studies targeting both eastern and western Washington *S. occidentalis* populations have not found substantial variation in physiological function, despite a warmer climate for the eastern populations^29,30^. Nonetheless, with the climate in the PNW expected to increase 0.1–0.6 °C per decade^68^, it is likely that more habitat will become suitable for *S. occidentalis*, thus promoting favorable conditions for expansion.

Extensive shoreline development and urbanization could limit future population expansion of *S. occidentalis*, even as the climate becomes warmer and more suitable habitat is available. Though we are unaware of any urban or suburban *S. occidentalis* populations in the Puget Sound region, they are relatively common in habitats surrounding the metropolitan areas of San Francisco and Los Angeles. Increasing coastal development around the Puget Sound threatens to further reduce genetic diversity within subpopulations. Although patterns are highly taxon specific, urban fragmentation typically leads to increased genetic drift within fragmented populations and reduced gene flow among them^69,70^. This suggests that the future of the northern *S. occidentalis* populations could be contingent on a balance between increased suitable habitat with climate change and decreased access to or destruction of this habitat with anthropogenic development. The threat of coastal development is especially pertinent as it frequently involves shoreline armoring in the form of sea walls, revetments, and bulkheads. Armoring effectively limits the beaching of driftwood and logs, reduces the presence of beach wrack and its associated invertebrates (a food source for *S. occidentalis*), and removes the cover of riparian vegetation^71^. Although no studies have directly examined impacts on lizard populations, armoring can have strong detrimental effects on species assemblages and abundance^72,73^. Fortunately, armor removal can effectively restore these key elements of shoreline habitats^74,75^.

Urban development may have already led to extirpation of local lizard assemblages, thus population reintroductions may become necessary. Historical museum records indicate that *S. occidentalis* occurred one mile south of Lincoln Park, Seattle. We were unable to detect *S. occidentalis* in Lincoln Park. However, the locality from which the specimens were found is now developed with little to no natural habitat remaining, so it is unlikely that the population has persisted. As coastal development in the Puget Sound region continues, more isolated groups may, or already have, become threatened. As such, expanded studies on the ecology and population demographics of the species in the region will prove useful for potential reintroductions. Our study provides important genetic information for guiding the selection of source populations to be used for reintroductions by demonstrating that unique alleles are present in many subpopulations across the Puget Sound region. One approach could be to translocate gravid females from a locality that shares the distinct alleles associated with the extirpated group. Another approach could utilize translocated individuals from multiple subpopulations to increase the genetic diversity of the reintroduced group, considering the low genetic diversity within any given subpopulations. Lastly, and especially pertinent for locally extirpated groups with an unknown genetic heritage (e.g., Lincoln Park), reintroduction efforts could utilize individuals from Chuckanut, considering that the assemblage at this locality is known to be human introduced.

Although a warming climate may lead to natural expansion of the Puget Sound population, occupying the northernmost extent of the distributional range exposes them to threats that the remainder of the species does not face. We provide evidence for a population that has undergone a relatively recent and expansive growth, which could indicate continued expansion as the climate becomes more favorable. However, multiple compounding factors limit this success. The pattern of colonization limited to highly specific stretches of south-facing coastal habitats with high sun exposure, including islands and peninsulas, has likely limited gene flow and accelerated genetic drift producing the genetic patterns described herein. Further, geographic discontinuity coupled with expanded urban development may impede the group’s ability to take advantage of the rapidly warming climate. The northernmost *S. occidentalis* population would be expected to continue a northward expansion, but the adaptive potential of the group may not be great enough to keep up with the rampant rate of anthropogenic change.

## Supplementary Information

Supplementary Information.

## Data Availability

DNA sequence data generated for this study are deposited at the NCBI Sequence Read Archive (SRA); accession numbers SAMN20963029—20963135. Datasets and R scripts used in the study are available on Dryad (10.5061/dryad.70rxwdc0f).
