# Supplementary material for: Population expansion, divergence, and persistence in Western Fence Lizards (Sceloporus occidentalis) at the northern extreme of their distributional range
Source: Sci Rep. 2022 Apr 15;12:6310. doi: 10.1038/s41598-022-10233-9 (PMC9012774; doi:10.1038/s41598-022-10233-9)
Supplement: Supplementary file 1 — Supplementary Information. [file 41598_2022_10233_MOESM1_ESM.pdf]

Population expansion, divergence, and persistence in Western Fence Lizards (*Sceloporus occidentalis*) at the northern extreme of their distributional range

Hayden R. Davis[1,*], **Simone Des Roches**[2], **Roger A. Anderson**[3], **and Adam D. Leaché**[1]

[1]Department of Biology & Burke Museum of Natural History and Culture, University of Washington, Seattle, WA 98195; USA
[2]School of Aquatic and Fisheries Sciences, University of Washington, Seattle, WA 98195; USA
[3]Department of Biology, Western Washington University, Bellingham, WA 98225; USA
[*]hrdavis1@uw.edu

ABSTRACT

Population dynamics within species at the edge of their distributional range, including the formation of genetic structure during range expansion, are difficult to study when they have had limited time to evolve. Western Fence Lizards (*Sceloporus occidentalis*) have a patchy distribution at the northern edge of their range around the Puget Sound, Washington, where they almost exclusively occur on imperiled coastal habitats. The entire region was covered by Pleistocene glaciation as recently as 16,000 years ago, suggesting that populations must have colonized these habitats relatively recently. We tested for population differentiation across this landscape using genome-wide SNPs and morphological data. A time-calibrated species tree supports the hypothesis of a post-glacial establishment and subsequent population expansion into the region. Despite a strong signal for fine-scale population genetic structure across the Puget Sound with as many as 8 — 10 distinct subpopulations supported by the SNP data, there is minimal evidence for morphological differentiation at this same spatiotemporal scale. Historical demographic analyses suggest that populations expanded and diverged across the region as the Cordilleran Ice Sheet receded. Population isolation, lack of dispersal corridors, and strict habitat requirements are the key drivers of population divergence in this system. These same factors may prove detrimental to the future persistence of populations as they cope with increasing shoreline development associated with urbanization.

Table of contents

Table S1. Voucher specimens used in the study.
Table S2. Summary statistics for the reference-based assembly of *S. occidentalis*.
Table S3. Specimens and partitioning scheme used in the time-calibrated species tree analysis using SNAPP.
Table S4. Specimens and morphological data used in morphological analyses.
Table S5. Summary statistics for morphological data.
Table S6. Summary statistics for morphological PCA.
Figure S1. Photographs of *Sceloporus occidentalis* habitats in the Puget Sound Region of Western Washington (A–F).
Figure S2. Photographs of *Sceloporus occidentalis* habitats in the Puget Sound Region of Western Washington (G–L).
Figure S3. Cross-validation scores for the ADMIXTURE analyses using 0% and 50% missing data datasets.
Figure S4. Discriminant analysis of principal components of genomic data.
Figure S5. Relatedness among samples calculated using the relatedness2 function in VCFtools.
Figure S6. Phylogenetic relationships estimated using RAxML with the concatenated data.
Figure S7. Goodness-of-fit test results for the demographic model.

Supplemental Table 1. Voucher specimens used in the study, detailed locality data, and NCBI Sequence Read Archive (SRA) accession numbers for the ddRADseq data. Samples sequenced in previous studies are labeled with an asterisk. Field number series correspond to “ADL” (Adam D. Leaché), “HRD” (Hayden R. Davis), “JAG’ (Jared A. Grummer), “RA” (Roger Anderson).

Date Collected	State	County	Location	Latitude	Longitude	Field Number	Voucher	NCBI SRA
May 2020	Washington	Island	Camano Island, Juniper Beach	48.2267615	-122.4149888	ADL 4955	UWBM:HERP:10047	SAMN20963049
May 2020	Washington	Island	Camano Island, Juniper Beach	48.2267615	-122.4149888	ADL 4956	UWBM:HERP:10048	SAMN20963050
May 2020	Washington	Island	Camano Island, Juniper Beach	48.2267615	-122.4149888	ADL 4957	UWBM:HERP:10049	SAMN20963051
May 2020	Washington	Island	Camano Island, Juniper Beach	48.2267615	-122.4149888	ADL 4958	UWBM:HERP:10050	SAMN20963052
August 2020	Washington	Jefferson	Beckett Point	48.077403	-122.883344	ADL 5007	UWBM:HERP:10099	SAMN20963053
August 2020	Washington	Jefferson	Beckett Point	48.077403	-122.883344	ADL 5008	UWBM:HERP:10100	SAMN20963054
August 2020	Washington	Jefferson	Beckett Point	48.077403	-122.883344	ADL 5009	UWBM:HERP:10101	SAMN20963055
August 2020	Washington	Jefferson	Beckett Point	48.077403	-122.883344	ADL 5010	UWBM:HERP:10102	SAMN20963056
August 2020	Washington	Jefferson	Duckabush, Dathne Ln.	47.652455	-122.926409	ADL 5012	UWBM:HERP:10104	SAMN20963057
August 2020	Washington	Jefferson	Duckabush, Dathne Ln.	47.652455	-122.926409	ADL 5013	UWBM:HERP:10105	SAMN20963058
August 2020	Washington	Jefferson	Duckabush, Mt. Jupiter Rd.	47.658081	-122.927216	ADL 5014	UWBM:HERP:10106	SAMN20963059
August 2020	Washington	Jefferson	Duckabush, Mt. Jupiter Rd.	47.658081	-122.927216	ADL 5015	UWBM:HERP:10107	SAMN20963060
August 2020	Washington	Jefferson	Port Townsend	48.141738	-122.756934	ADL 5003	UWBM:HERP:10095	SAMN20963061
August 2020	Washington	Jefferson	Port Townsend	48.141738	-122.756934	ADL 5004	UWBM:HERP:10096	SAMN20963062
August 2020	Washington	Jefferson	Port Townsend	48.141738	-122.756934	ADL 5005	UWBM:HERP:10097	SAMN20963063
August 2020	Washington	Jefferson	Port Townsend	48.141738	-122.756934	ADL 5006	UWBM:HERP:10098	SAMN20963064
April 2020	Washington	King	Burien, Marine View Park	47.412711	-122.342353	ADL 4951	UWBM:HERP:10043	SAMN20963065
April 2020	Washington	King	Burien, Marine View Park	47.412711	-122.342353	ADL 4952	UWBM:HERP:10044	SAMN20963066
April 2020	Washington	King	Burien, Marine View Park	47.412711	-122.342353	ADL 4954	UWBM:HERP:10046	SAMN20963067
July 2020	Washington	King	Maury Island, beach below bluffs trailhead	47.36802	-122.43324	ADL 4964	UWBM:HERP:10056	SAMN20963068
July 2020	Washington	King	Maury Island, Marine View Park trailhead	47.38183	-122.40844	ADL 4959	UWBM:HERP:10051	SAMN20963069
July 2020	Washington	King	Maury Island, Marine View Park trailhead	47.38183	-122.40844	ADL 4960	UWBM:HERP:10052	SAMN20963070
July 2020	Washington	King	Maury Island, Marine View Park trailhead	47.38183	-122.40844	ADL 4961	UWBM:HERP:10053	SAMN20963071
July 2020	Washington	King	Maury Island, Marine View Park trailhead	47.38183	-122.40844	ADL 4962	UWBM:HERP:10054	SAMN20963072
August 2020	Washington	Kitsap	Holly, Anderson Cove, NW Old Holly Hill Rd.	47.570287	-122.971033	ADL 4995	UWBM:HERP:10087	SAMN20963073
August 2020	Washington	Kitsap	Holly, Anderson Cove, NW Old Holly Hill Rd.	47.570287	-122.971033	ADL 4996	UWBM:HERP:10088	SAMN20963074
August 2020	Washington	Kitsap	Holly, Anderson Cove, NW Old Holly Hill Rd.	47.570287	-122.971033	ADL 4997	UWBM:HERP:10089	SAMN20963075
August 2020	Washington	Kitsap	Holly, Anderson Cove, NW Old Holly Hill Rd.	47.570287	-122.971033	ADL 4998	UWBM:HERP:10090	SAMN20963076
June 2016	Washington	Klickitat	Columbia River Gorge	45.71012	-121.31915	RA L63	N/A	SAMN20963077
June 2016	Washington	Klickitat	Columbia River Gorge	45.71012	-121.31915	RA X63	N/A	SAMN20963078
June 2016	Washington	Klickitat	Columbia River Gorge	45.71012	-121.31915	RA R82	N/A	SAMN20963079
June 2016	Washington	Klickitat	Columbia River Gorge	45.71012	-121.31915	RA A26	N/A	SAMN20963080
August 2020	Washington	Mason	Belfair, NE Gladwin Rd.	47.4310891	-122.8699313	ADL 4976	UWBM:HERP:10068	SAMN20963081
August 2020	Washington	Mason	Belfair, NE Gladwin Rd.	47.4310891	-122.8699313	ADL 4977	UWBM:HERP:10069	SAMN20963082
August 2020	Washington	Mason	NE Dewatto Holly Rd.	47.458181	-123.029584	ADL 4989	UWBM:HERP:10081	SAMN20963083
August 2020	Washington	Mason	NE Dewatto Holly Rd.	47.458181	-123.029584	ADL 4990	UWBM:HERP:10082	SAMN20963084
August 2020	Washington	Mason	Tahuya, Ayres "Bald" Point	47.3741942	-123.1110878	ADL 4984	UWBM:HERP:10076	SAMN20963085
August 2020	Washington	Mason	Tahuya, Ayres "Bald" Point	47.3741942	-123.1110878	ADL 4985	UWBM:HERP:10077	SAMN20963086
August 2020	Washington	Mason	Tahuya, Ayres "Bald" Point	47.3741942	-123.1110878	ADL 4986	UWBM:HERP:10078	SAMN20963087
August 2020	Washington	Mason	Tahuya, Ayres "Bald" Point	47.3741942	-123.1110878	ADL 4987	UWBM:HERP:10079	SAMN20963088
August 2020	Washington	Mason	Tree Farm, NE Dewatto Holly Rd.	47.513872	-122.979099	ADL 4991	UWBM:HERP:10083	SAMN20963089
August 2020	Washington	Mason	Tree Farm, NE Dewatto Holly Rd.	47.513872	-122.979099	ADL 4992	UWBM:HERP:10084	SAMN20963090
August 2020	Washington	Mason	Tree Farm, NE Dewatto Holly Rd.	47.513872	-122.979099	ADL 4993	UWBM:HERP:10085	SAMN20963091
August 2020	Washington	Mason	Tree Farm, NE Dewatto Holly Rd.	47.513872	-122.979099	ADL 4994	UWBM:HERP:10086	SAMN20963092
August 2020	Washington	Pierce	Anderson Island, Andy’s Marine Park	47.14693	-122.73322	HRD 001	UWBM:HERP:10108	SAMN20963093
August 2020	Washington	Pierce	Anderson Island, Andy’s Marine Park	47.14693	-122.73322	HRD 002	UWBM:HERP:10109	SAMN20963094
August 2020	Washington	Pierce	Anderson Island, Andy’s Marine Park	47.14693	-122.73322	HRD 003	UWBM:HERP:10110	SAMN20963095
August 2020	Washington	Pierce	Anderson Island, Andy’s Marine Park	47.14693	-122.73322	HRD 004	UWBM:HERP:10111	SAMN20963096
April 2020	Washington	Pierce	Cambers Creek Regional Park, Shoreline	47.19992	-122.583072	ADL 4947	UWBM:HERP:10039	SAMN20963097
April 2020	Washington	Pierce	Cambers Creek Regional Park, Shoreline	47.19992	-122.583072	ADL 4948	UWBM:HERP:10040	SAMN20963098
April 2020	Washington	Pierce	Cambers Creek Regional Park, Shoreline	47.19992	-122.583072	ADL 4950	UWBM:HERP:10042	SAMN20963099
April 2020	Washington	Pierce	Cambers Creek Regional Park, Tunnel	47.201528	-122.574266	ADL 4942	UWBM:HERP:10034	SAMN20963100
April 2020	Washington	Pierce	Cambers Creek Regional Park, Tunnel	47.201528	-122.574266	ADL 4943	UWBM:HERP:10035	SAMN20963101
April 2020	Washington	Pierce	Cambers Creek Regional Park, Tunnel	47.201528	-122.574266	ADL 4944	UWBM:HERP:10036	SAMN20963102
August 2020	Washington	Pierce	Joemma Beach State Park	47.2254645	-122.809815	ADL 4999	UWBM:HERP:10091	SAMN20963103
August 2020	Washington	Pierce	Joemma Beach State Park	47.2254645	-122.809815	ADL 5000	UWBM:HERP:10092	SAMN20963104
August 2020	Washington	Pierce	Joemma Beach State Park	47.2254645	-122.809815	ADL 5001	UWBM:HERP:10093	SAMN20963105
August 2020	Washington	Pierce	Joemma Beach State Park	47.2254645	-122.809815	ADL 5002	UWBM:HERP:10094	SAMN20963106
August 2020	Washington	Pierce	Ketron Island, South end	47.1472211	-122.6390565	ADL 4978	UWBM:HERP:10070	SAMN20963107
August 2020	Washington	Pierce	Ketron Island, South end	47.1472211	-122.6390565	ADL 4981	UWBM:HERP:10073	SAMN20963108
August 2020	Washington	Pierce	Ketron Island, South end	47.1472211	-122.6390565	ADL 4982	UWBM:HERP:10074	SAMN20963109
August 2020	Washington	Pierce	Ketron Island, South end	47.1472211	-122.6390565	ADL 4983	UWBM:HERP:10075	SAMN20963110
July 2020	Washington	Pierce	Tacoma, Point Defiance Park	47.30728	-122.5382	ADL 4967	UWBM:HERP:10059	SAMN20963111
July 2020	Washington	Pierce	Tacoma, Point Defiance Park	47.30728	-122.5382	ADL 4968	UWBM:HERP:10060	SAMN20963112
July 2020	Washington	Pierce	Tacoma, Point Defiance Park	47.30728	-122.5382	ADL 4969	UWBM:HERP:10061	SAMN20963113
August 2020	Washington	Pierce	Wauna, Purdy Sand Spit Park	47.3806498	-122.6377161	ADL 4972	UWBM:HERP:10064	SAMN20963114
August 2020	Washington	Pierce	Wauna, Purdy Sand Spit Park	47.3806498	-122.6377161	ADL 4973	UWBM:HERP:10065	SAMN20963115
August 2020	Washington	Pierce	Wauna, Purdy Sand Spit Park	47.3806498	-122.6377161	ADL 4974	UWBM:HERP:10066	SAMN20963116
August 2020	Washington	Pierce	Wauna, Purdy Sand Spit Park	47.3806498	-122.6377161	ADL 4975	UWBM:HERP:10067	SAMN20963117
N/A	Washington	Snohomish	Spee Bi Dah, West of Marysville	48.09348	-122.32675	RA W6	N/A	SAMN20963118
August 2016	Washington	Snohomish	Spee Bi Dah, West of Marysville	48.09348	-122.32675	RA WW29	N/A	SAMN20963119
September 2011	Washington	Snohomish	Tulare Beach	48.105321	-122.344886	ADL 4127	UWBM:HERP:6275	SAMN20963120
September 2011	Washington	Snohomish	Tulare Beach	48.105321	-122.344886	ADL 4128	UWBM:HERP:6276	SAMN20963121
September 2011	Washington	Snohomish	Tulare Beach	48.105321	-122.344886	ADL 4129	UWBM:HERP:6277	SAMN20963122
September 2011	Washington	Snohomish	Tulare Beach	48.105321	-122.344886	ADL 4130	UWBM:HERP:6278	SAMN20963123
September 2011	Washington	Snohomish	Tulare Beach	48.105321	-122.344886	ADL 4131	UWBM:HERP:6279	SAMN20963124
September 2011	Washington	Snohomish	Tulare Beach	48.105321	-122.344886	ADL 4132	UWBM:HERP:6280	SAMN20963125
September 2011	Washington	Snohomish	Tulare Beach	48.105321	-122.344886	ADL 4133	UWBM:HERP:6281	SAMN20963126
May 2016	Washington	Whatcom	Chuckanut Drive	48.692863	-122.495858	RA WW27	N/A	SAMN20963127
May 2016	Washington	Whatcom	Chuckanut Drive	48.692863	-122.495858	RA W24	N/A	SAMN20963128
May 2016	Washington	Whatcom	Chuckanut Drive	48.692863	-122.495858	RA X57	N/A	SAMN20963129
May 2016	Washington	Whatcom	Chuckanut Drive	48.692863	-122.495858	RA W47	N/A	SAMN20963130
October 2012	Washington	Chelan	Swakane; 50 m from Swakane Canyon road	47.565	-120.3206	RA X110	UWBM:HERP:7613	SAMN20963131
October 2012	Washington	Chelan	Swakane; 50 m from Swakane Canyon road	47.565	-120.3206	RA L57	UWBM:HERP:7614	SAMN20963132
October 2012	Washington	Chelan	Swakane; 50 m from Swakane Canyon road	47.565	-120.3206	RA L32	UWBM:HERP:7615	SAMN20963133
October 2012	Washington	Chelan	Swakane; 50 m from Swakane Canyon road	47.565	-120.3206	RA L59	UWBM:HERP:7616	SAMN20963134
October 2012	Washington	Chelan	Swakane; 50 m from Swakane Canyon road	47.565	-120.3206	RA A15	UWBM:HERP:7617	SAMN20963135
May 2012	Washington	Chelan	Leavenworth	47.589	-120.676	JAG 001	UWBM:HERP:7976	SAMN14488721*
N/A	Washington	Yakima	Tieton River, Oak Creek Road	46.7277	-120.8275	YAK1	N/A	SAMN14488785*
N/A	Washington	Yakima	Tieton River, Oak Creek Road	46.7277	-120.8275	YAK3	N/A	SAMN14488786*
N/A	Oregon	Jefferson	Skunk Hollow	44.4075	-121.0729	SKK15	N/A	SAMN14488759*
N/A	Oregon	Jefferson	Skunk Hollow	44.4075	-121.0729	SKK18	N/A	SAMN14488760*
N/A	Oregon	Wasco	Shaniko Jct. Rest Area	44.8948	-120.9357	SHN7	N/A	SAMN14488758*
N/A	Oregon	Wasco	Shaniko Jct. Rest Area	44.8948	-120.9357	SHN15	N/A	SAMN14488757*
N/A	Oregon	Josephine	Selma	42.2953	-123.741	SLM1	N/A	SAMN14488764*
N/A	Oregon	Josephine	Selma	42.2953	-123.741	SLM3	N/A	SAMN14488765*

Supplemental Table 2. Summary statistics for the reference-based assembly of *S. occidentalis*. The number of loci in the final assembly ("loci_in_assembly") is based on 50% missing data. The assembly with 0% missing data resulted in 1,037 loci per sample.

Sample	reads_raw	reads_passed_filter	refseq_mapped_reads	clusters_total	avg_depth	hetero_est	error_est	loci_in_assembly
ADL4974_wauna	788,562	787,391	644,780	14,076	45.8	0.0029	0.0005	2,826
ADL4975_wauna	1,445,622	1,443,378	1,185,484	51,444	23.0	0.0031	0.0003	3,115
ADL4976_belfair	2,454,000	2,451,469	1,966,149	21,129	93.1	0.0036	0.0002	3,022
ADL4977_belfair	1,039,443	1,038,217	834,596	19,922	41.9	0.0029	0.0004	2,752
ADL4978_ketron	3,658,106	3,653,435	2,967,882	55,486	53.5	0.0042	0.0003	3,301
ADL4981_ketron	3,184,947	3,181,345	2,555,642	19,176	133.3	0.0045	0.0003	3,264
ADL4982_ketron	1,516,708	1,514,227	1,210,772	41,267	29.3	0.0032	0.0003	2,816
ADL4983_ketron	2,958,616	2,954,924	2,427,740	38,997	62.3	0.0028	0.0002	3,253
ADL4942_cambers	433,834	432,791	349,516	14,343	24.4	0.0022	0.0007	2,846
ADL4943_cambers	1,912,034	1,910,076	1,581,559	16,578	95.4	0.0078	0.0002	3,236
ADL4944_cambers	1,469,402	1,466,429	1,207,638	16,489	73.2	0.0031	0.0004	3,253
ADL4947_cambers	1,740,618	1,735,127	1,445,377	56,103	25.8	0.0036	0.0003	3,242
ADL4948_cambers	1,059,763	1,058,724	865,360	8,540	101.3	0.0069	0.0003	3,047
ADL4950_cambers	944,191	943,260	774,868	12,045	64.3	0.0030	0.0003	2,762
ADL4951_burien	545,353	544,894	445,627	6,477	68.8	0.0018	0.0006	2,575
ADL4952_burien	1,142,715	1,141,367	945,226	22,038	42.9	0.0022	0.0004	3,148
ADL4954_burien	419,980	419,498	339,837	6,899	49.3	0.0017	0.0006	2,368
ADL4955_camano	556,865	555,604	444,353	12,337	36.0	0.0031	0.0006	2,279
ADL4956_camano	3,105,538	3,100,695	2,510,263	18,099	138.7	0.0038	0.0003	3,198
ADL4957_camano	2,088,062	2,085,046	1,677,250	19,247	87.1	0.0039	0.0003	2,752
ADL4958_camano	643,991	643,080	516,995	9,124	56.7	0.0018	0.0005	2,477
ADL4959_maury	499,507	498,977	403,497	6,708	60.2	0.0018	0.0006	2,545
ADL4960_maury	1,552,872	1,548,055	1,283,463	53,539	24.0	0.0025	0.0003	3,314
ADL4961_maury	424,327	423,752	342,498	9,173	37.3	0.0020	0.0006	2,298
ADL4962_maury	3,965,164	3,960,984	3,293,535	28,151	117.0	0.0035	0.0003	3,390
ADL4964_maury	3,039,217	3,036,290	2,515,542	18,795	133.8	0.0033	0.0002	3,345
ADL4967_ptdefiance	928,366	927,224	763,045	11,181	68.2	0.0026	0.0004	3,166
ADL4968_ptdefiance	316,662	316,242	256,884	6,151	41.8	0.0020	0.0006	2,406
ADL4969_ptdefiance	2,598,760	2,594,321	2,148,195	20,507	104.8	0.0034	0.0003	3,395
ADL4972_wauna	237,436	232,185	188,053	29,480	6.4	0.0023	0.0007	2,203
ADL4973_wauna	1,078,782	1,075,555	892,683	19,798	45.1	0.0059	0.0004	3,168
ADL4984_tahuya	1,220,584	1,216,254	997,463	43,845	22.8	0.0077	0.0003	3,161
ADL4985_tahuya	1,642,027	1,640,042	1,364,801	29,303	46.6	0.0029	0.0003	3,273
ADL4986_tahuya	819,490	818,878	667,938	9,063	73.7	0.0032	0.0004	2,275
ADL4987_tahuya	3,311,448	3,308,614	2,715,338	15,458	175.7	0.0037	0.0003	3,356
ADL4989_dewatto	1,498,687	1,497,262	1,244,818	17,611	70.7	0.0023	0.0003	3,243
ADL4990_dewatto	2,166,476	2,164,138	1,775,703	16,000	111.0	0.0027	0.0003	3,317
ADL4991_treefarm	2,290,832	2,288,579	1,890,719	12,290	153.8	0.0030	0.0002	3,332
ADL4992_treefarm	625,860	624,964	511,827	17,870	28.6	0.0029	0.0005	2,780
ADL4993_treefarm	1,079,890	1,077,657	881,789	21,668	40.7	0.0041	0.0004	2,982
ADL4994_treefarm	2,269,414	2,266,826	1,853,661	12,370	149.9	0.0037	0.0003	3,302
ADL4995_holly	1,122,801	1,120,991	930,095	37,171	25.0	0.0025	0.0003	2,981
ADL4996_holly	2,231,463	2,229,470	1,844,294	13,173	140.0	0.0024	0.0002	3,333
ADL4997_holly	432,621	431,865	356,648	7,555	47.2	0.0032	0.0006	1,901
ADL4998_holly	2,199,926	2,197,133	1,799,652	23,591	76.3	0.0036	0.0003	3,223
ADL4999_joemma	1,667,418	1,662,295	1,379,965	94,837	14.6	0.0040	0.0003	3,210
ADL5000_joemma	1,363,351	1,362,180	1,128,077	13,241	85.2	0.0044	0.0004	2,734
ADL5001_joemma	366,957	366,474	301,628	7,011	43.0	0.0022	0.0007	1,836
ADL5002_joemma	642,085	641,275	530,341	8,715	60.9	0.0042	0.0006	2,118
ADL5003_townsend	5,448,820	5,443,401	4,539,374	15,962	284.4	0.0039	0.0003	3,383
ADL5004_townsend	2,128,740	2,126,823	1,779,135	28,402	62.6	0.0034	0.0002	3,269
ADL5005_townsend	3,238,873	3,234,630	2,701,614	24,056	112.3	0.0031	0.0003	3,382
ADL5006_townsend	3,289,664	3,285,529	2,763,867	44,719	61.8	0.0030	0.0002	3,385
ADL5007_beckett	987,698	985,934	816,504	19,998	40.8	0.0023	0.0004	2,855
ADL5008_beckett	1,150,801	1,149,212	948,127	17,456	54.3	0.0030	0.0004	2,879
ADL5009_beckett	1,541,719	1,538,214	1,286,838	48,201	26.7	0.0030	0.0003	3,032
ADL5010_beckett	844,655	843,644	707,802	16,705	42.4	0.0022	0.0004	2,678
ADL5012_duckabush	1,806,558	1,803,584	1,491,303	63,284	23.6	0.0024	0.0003	3,093
ADL5013_duckabush	2,261,447	2,258,997	1,848,208	17,581	105.1	0.0033	0.0003	3,243
ADL5014_duckabush	912,584	910,614	734,324	12,408	59.2	0.0022	0.0004	3,093
ADL5015_duckabush	665,671	664,152	524,729	17,543	29.9	0.0022	0.0006	2,914
HERP6275_tulare	1,622,062	1,619,876	1,343,574	15,671	85.7	0.0025	0.0003	3,350
HERP6276_tulare	319,880	319,311	247,032	10,723	23.0	0.0021	0.0007	2,628
HERP6277_tulare	430,169	427,497	354,062	14,159	25.0	0.0017	0.0006	2,581
HERP6278_tulare	564,723	558,062	456,879	42,956	10.6	0.0024	0.0006	2,681
HERP6279_tulare	304,613	293,392	232,616	31,338	7.4	0.0021	0.0007	2,324
HERP6280_tulare	1,647,969	1,624,908	1,351,996	113,929	11.9	0.0038	0.0004	3,235
HERP6281_tulare	175,871	170,054	129,177	7,911	16.3	0.0023	0.0008	1,781
HRD001_anderson	911,830	910,571	762,668	48,185	15.8	0.0022	0.0004	2,876
HRD002_anderson	2,313,203	2,310,816	1,932,133	35,247	54.8	0.0028	0.0002	3,343
HRD003_anderson	526,736	525,281	436,007	27,609	15.8	0.0019	0.0006	2,410
HRD004_anderson	1,054,692	1,053,385	875,851	36,683	23.9	0.0028	0.0004	3,103
RA_W24_chuckanut	844,245	843,007	685,028	11,921	57.5	0.0020	0.0004	2,646
RA_W47_chuckanut	581,456	580,631	475,767	12,802	37.2	0.0017	0.0005	2,427
RA_W6_speedibah	2,054,685	2,051,955	1,655,405	12,172	136.0	0.0030	0.0002	2,751
RA_WW27_chuckanut	1,045,699	1,043,978	833,649	13,171	63.3	0.0026	0.0004	3,063
RA_WW29_speedibah	2,451,777	2,448,562	1,949,787	15,357	127.0	0.0045	0.0002	2,921
RA_X57_chuckanut	861,777	859,796	707,117	16,628	42.5	0.0064	0.0005	2,233

Supplemental Table 3. Specimens and partitioning scheme used in the time-calibrated species tree analysis using SNAPP.

Sample	Location	Partition name
UWBM:HERP:10049	Camano Island	Puget Sound
UWBM:HERP:10101	Beckett Point	Puget Sound
UWBM:HERP:10105	Duckabush	Puget Sound
UWBM:HERP:10095	Port Townsend	Puget Sound
UWBM:HERP:10043	Burien	Puget Sound
UWBM:HERP:10088	Holly	Puget Sound
UWBM:HERP:10077	Tahuya	Puget Sound
UWBM:HERP:10109	Anderson Island	Puget Sound
UWBM:HERP:10035	Chambers Creek	Puget Sound
UWBM:HERP:10061	Point Defiance	Puget Sound
UWBM:HERP:10067	Wauna	Puget Sound
UWBM:HERP:7976	Leavenworth	North Cascades
UWBM:HERP:7615	Swakane	North Cascades
UWBM:HERP:7616	Swakane	North Cascades
RA A26	Columbia River	Columbia River
RA R82	Columbia River	Columbia River
YAK1	Tieton River, Oak Creek Road	Yakima
YAK3	Tieton River, Oak Creek Road	Yakima
SLM1	Oregon: Selma	Selma
SLM3	Oregon: Selma	Selma
SHN15	Oregon: Shaniko	Shaniko
SHN7	Oregon: Shaniko	Shaniko
SKK15	Oregon: Skunk Hollow	Skunk Hollow
SKK18	Oregon: Skunk Hollow	Skunk Hollow

Supplemental Table 4. Specimens and morphological data used for morphological analyses. Character acronyms detailed in Materials and Methods. svl_live is not mentioned in the Materials and Methods, but we have included here to account for size changes due to preservation.

id	sex	pop	svl_live	tl	svl	hl	hw	lfl	rfl	rtl	ltl	lfp	rfp	ltl	rtl	ms	ds
ADL_4942	m	TAC	70	67	67	13.6	14	18.5	18.5	12	12.5	13	13	21	21	7	43
ADL_4943	m	TAC	68	83	63.5	13	13.8	17.7	17.9	12.5	12	15	14	20	21	8	39
ADL_4944	f	TAC	71	61	66	13.1	13.9	17.8	18.4	11.5	12.5	14	13	20	20	9	43
ADL_4945	m	TAC	77	40	73.5	12.1	14.8	18.9	19.4	13	12	13	13	22	21	8	43
ADL_4946	m	TAC	75	39	72	14.3	15.4	18.9	19.4	12.5	12	14	14	21	20	7	43
ADL_4947	m	TAC	68	80	63	13.1	13.2	17.3	17	13	12	13	13	19	19	7	43
ADL_4948	m	TAC	68	83	64.5	13.9	13.5	17.4	18.3	12	13	14	15	22	21	7	39
ADL_4949	m	TAC	64	78	60.5	12.6	13	17	16.6	12	11.5	14	15	21	23	6	41
ADL_4950	f	TAC	78	88	73.5	14.4	13.9	19.1	19.2	13.5	13	14	14	21	21	9	44
ADL_4951	m	TAC	75	74	71	13.6	14.6	19	19.5	12	12	13	15	19	20	8	43
ADL_4952	m	TAC	68	62	64	13.5	13.5	17.1	17.4	11	11.5	14	14	21	21	8	39
ADL_4953	m	TAC	65	77	61	12.6	12.3	17.7	17.6	11.5	12.5	13	13	21	21	9	42
ADL_4954	f	TAC	64	66	60	12.3	11.9	16.2	16	11	11	14	14	21	21	9	42
ADL_4955	m	TUL	67	87	65.5	13.2	13.1	17.7	17.8	12.5	12	15	15	21	21	9	41
ADL_4956	m	TUL	66	86	63.5	12.5	13.6	18.7	18.3	12.5	13	17	16	22	21	9	41
ADL_4957	m	TUL	68	62	66	13	13.7	17.6	18.8	12	12.5	15	16	21	22	9	36
ADL_4958	f	TUL	69	80	64	12.3	12.4	17.4	17.4	12.5	12.5	16	17	20	20	8	43
ADL_4959	m	TAC	61	65	53	12.7	12.1	16.1	16.4	11	11	14	14	21	21	9	41
ADL_4960	m	TAC	65	46	62.5	13.2	13.2	16.9	15.4	11.5	11.5	13	14	21	21	7	43
ADL_4961	m	TAC	66	43	65	13.6	13.5	17.4	18.1	12	12	15	14	20	19	7	41
ADL_4962	m	TAC	59	67	56	12.3	12	16.2	15.7	10.5	10	15	15	21	21	7	40
ADL_4963	m	TAC	69	47	66.5	13.8	13.9	17.3	17.5	12	12	14	14	20	20	8	42
ADL_4964	f	TAC	67	75	63	12.7	13.5	16.6	17.1	12	12	14	14	21	20	8	43
ADL_4965	m	TAC	53	63	50	11.1	10.7	12.9	13.8	10	10	15	14	23	22	9	45
ADL_4966	m	TAC	48	58	47	10.5	9.9	12.9	12.7	9	9	15	15	20	21	10	40
ADL_4967	m	TAC	69	81	66	14.2	14.3	18.9	18.6	12.5	NA	14	15	NA	21	8	42
ADL_4968	m	TAC	63	74	60	12.9	12.9	16.9	16.1	11.5	11	14	16	20	21	8	41
ADL_4969	f	TAC	70	75	68.5	13.7	13.6	17.9	17.6	11.5	11.5	12	14	24	22	10	43
ADL_4970	f	TAC	68	53	62.5	13.8	13.5	17.8	17.5	11	10.5	NA	NA	22	20	13	40
ADL_4972	m	JOE	65	57	64.5	13.4	13.6	18.5	18.2	12.5	12.5	14	12	20	20	10	43
ADL_4973	m	JOE	64	65	62.5	13.4	13.8	17.8	18.1	12.5	12.5	13	14	21	21	8	43
ADL_4974	m	JOE	63	37	62.5	13.3	13.5	17.5	16.8	11.5	11.5	14	13	20	21	9	42
ADL_4975	f	JOE	68	59	67.5	13.3	13.5	17.8	17.9	12	12.5	13	14	21	21	10	45
ADL_4976	m	TAC	67	79	65	13.6	13.9	18.1	18.1	12	11.5	15	14	19	20	9	41
ADL_4977	m	TAC	67	55	64.5	13.5	14.4	17.7	18.7	11.5	12	12	13	21	20	9	39
ADL_4978	f	TAC	55	69	53.5	11	10.8	13.8	14.1	11.5	11	14	14	21	21	8	43
ADL_4981	m	TAC	69	83	67	14.1	13.6	17.4	18	12.5	13.5	14	14	22	23	6	44
ADL_4982	m	TAC	62	61	59.5	13	12.9	16.2	16.4	12	12.5	16	16	24	23	7	43
ADL_4983	m	TAC	58	77	56	12.1	11.9	16	15.7	12.5	12	15	15	23	24	7	43
ADL_4984	m	TAH	74	58	70	14	15.3	19.5	19.6	13.5	12.5	13	15	21	21	9	39
ADL_4985	m	TAH	72	84	69.5	14.1	15.8	19.8	19.4	12.5	12	14	13	20	20	7	39
ADL_4986	f	TAH	80	39	78	15.3	16.2	20.8	21.2	13.5	13.5	13	14	20	19	10	38
ADL_4987	f	TAH	65	69	61	12.4	13.7	17.9	16.5	11	11	14	13	20	21	9	37
ADL_4989	m	TAH	66	37	62.5	13.2	13.4	17.7	17.8	12	12	14	14	19	21	9	40
ADL_4990	f	TAH	75	80	73.5	14.7	14.8	19.4	19.7	13.5	13	13	13	20	20	10	41
ADL_4991	f	TAH	75	80	72	14	14.8	18.9	19.3	12	12	14	14	21	20	7	42
ADL_4992	f	TAH	72	67	69	13.8	13.7	19.2	18.3	11.5	11.5	15	15	21	21	7	40
ADL_4993	m	TAH	71	65	67	NA	NA	18.1	18.7	NA	NA	NA	NA	NA	NA	NA	NA
ADL_4994	m	TAH	71	66	68.5	14	14.9	19	18.4	12.5	12.5	15	14	21	22	7	39
ADL_4995	m	TAH	67	80	64.5	13.3	13.7	17.4	17.3	11.5	11.5	14	13	20	21	8	40
ADL_4996	m	TAH	67	48	65.5	13.5	13.5	19.7	18.8	12	12	14	14	20	20	9	41
ADL_4997	m	TAH	60	75	57.5	12	12.5	16	16.2	11	11	16	15	21	20	8	41
ADL_4998	m	TAH	55	67	53.5	11.5	11.5	14.8	14.8	11	10	14	14	20	19	9	40
ADL_4999	m	JOE	68	31	63.5	13.4	13.8	17.8	18.5	11	11.5	15	15	23	22	7	38
ADL_5000	m	JOE	74	90	71	14.1	14.8	18.9	19.4	12	12	15	15	18	20	7	42
ADL_5001	f	JOE	67	75	65.5	13	14.3	17.9	17.1	12	11.5	15	13	21	21	10	41
ADL_5002	f	JOE	52	63	50	11.1	11.1	13.9	13.6	10	10	14	13	19	18	7	40
ADL_5003	m	JOE	72	46	68.5	14	14.5	19.6	19.4	12.5	12	15	14	21	21	9	45
ADL_5004	m	JOE	70	88	66	13.7	14.2	19.3	18.3	13	13	15	15	20	21	10	42
ADL_5005	m	JOE	69	71	67	14.2	14.3	19	18.2	13	12	13	15	21	22	10	42
ADL_5006	m	JOE	64	78	61	12.4	12.9	17.6	18.3	12	12.5	14	14	23	21	9	41
ADL_5007	m	BKT	67	80	65.5	14.6	14	17.2	17.3	11	10.5	14	14	20	22	10	40
ADL_5008	m	BKT	69	86	66	14.2	14	18.4	17.8	12	12	16	13	20	18	8	42
ADL_5009	f	BKT	70	80	67	13.6	13.6	17.6	17.3	11.5	11	16	13	21	19	8	41
ADL_5010	f	BKT	69	77	67.5	13.8	13.4	17.5	18.1	11.5	11	12	14	20	19	10	44
ADL_5012	m	BKT	59	65	56.5	11.7	11.6	16.4	16	11	11	13	14	21	22	8	41
ADL_5013	m	BKT	25	26	24	NA	NA	NA	NA	NA	NA	NA	NA	NA	NA	NA	NA
ADL_5014	f	BKT	72	77	69	13.6	14.1	18.7	18.9	12	11.5	13	13	21	20	10	41
ADL_5015	f	BKT	64	50	61.5	12.2	13.4	16.9	16.7	11	11	13	14	19	21	8	39
HRD_001	m	TAC	61	78	59	12.1	12.3	16.4	16.5	12	12	14	16	20	20	8	40
HRD_002	f	TAC	76	84	62	14.6	15.2	18.7	20.6	12	12	14	13	22	23	9	42
HRD_003	f	TAC	65	75	62.5	12.5	12.6	16.8	17.3	11	11	13	14	20	21	8	45
HRD_004	f	TAC	65	35	62.5	13.2	13.5	16.9	16.9	11	11.5	14	15	20	20	9	41
ADL_4127	m	TUL	69	NA	67	14.3	13.8	18.1	18.4	NA	12.5	14	14	20	NA	7	41
ADL_4128	m	TUL	69	NA	65.5	13.4	13.3	17.7	16.7	12	11.5	14	13	22	18	7	41
ADL_4129	m	TUL	55	NA	54	11.6	11.2	13.7	14.3	11	10.5	14	14	20	23	8	38
ADL_4130	m	TUL	53	NA	50.5	11.1	11.3	14.2	13.8	10.5	9.5	14	15	22	22	8	42
ADL_4131	m	TUL	58	NA	55	11.4	11.9	14.7	15.3	11	11	13	13	21	20	7	40
ADL_4132	f	TUL	70	NA	65.5	12.8	13.6	17.6	17.4	12	11	14	15	20	19	7	42
ADL_4133	f	TUL	60	NA	57	11.9	11.4	15.6	15	11	11	14	15	21	19	9	40

Supplemental Table 5. Morphological summary statistics for Puget Sound *Sceloporus occidentalis* subpopulations.

	Population	SVL	HL	HW	LFL	RFL	LTL	RTL	LFP	RFP	LTL	RTL	MS	DS
	OLY	56.5	11.7	11.6	16.4	16	11	10.5	12	13	19	18	8	39
	KITs	50	11.1	11.1	13.9	13.6	10	10	13	12	18	18	7	38
**Minimum**	PUGs	47	10.5	9.9	12.9	12.7	9	9	12	13	19	19	6	39
	KITw	53.5	11.5	11.5	14.8	14.8	11	10	13	13	19	19	7	37
	PUGn	50.5	11.1	11.2	13.7	13.8	10.5	9.5	13	13	20	18	7	36
	OLY	69	14.6	14.1	18.7	18.9	12	12	16	14	21	22	10	44
	KITs	71	14.2	14.8	19.6	19.4	13	13	15	15	23	22	10	45
**Maximum**	PUGs	73.5	14.6	15.4	19.1	20.6	13.5	13.5	16	16	24	24	10	45
	KITw	78	15.3	16.2	20.8	21.2	13.5	13.5	16	15	21	22	10	42
	PUGn	66	13.4	13.7	18.7	18.8	12.5	13	17	17	22	23	9	43
	OLY	64.71	13.39	13.44	17.53	17.44	11.43	11.14	13.86	13.57	20.29	20.14	8.86	41.14
	KITs	64.13	13.28	13.69	17.97	17.82	12.00	11.96	14.17	13.92	20.67	20.75	8.83	42.00
**Mean**	PUGs	62.50	12.98	13.16	17.02	17.21	11.71	11.71	13.94	14.18	20.97	21.00	8.03	41.94
	KITw	66.54	13.52	14.14	18.47	18.25	12.12	11.88	14.08	13.92	20.31	20.38	8.38	39.77
	PUGn	60.65	12.32	12.55	16.49	16.48	11.70	11.45	14.60	14.90	21.00	20.50	8.10	40.40
	OLY	4.31	1.05	0.86	0.81	0.94	0.45	0.48	1.57	0.53	0.76	1.57	1.07	1.57
	KITs	5.28	0.85	0.97	1.47	1.53	0.85	0.78	0.83	1.00	1.44	1.06	1.27	1.95
**Standard Deviation**	PUGs	6.19	0.96	1.24	1.52	1.72	0.88	0.90	0.90	0.88	1.26	1.17	1.05	1.69
	KITw	6.73	1.06	1.32	1.67	1.72	0.94	0.92	0.86	0.76	0.63	0.87	1.12	1.36
	PUGn	5.88	0.80	1.03	1.77	1.75	0.75	1.07	1.17	1.29	0.82	1.58	0.88	2.07

Supplemental Table 6. Standard deviation, eigen values, and loadings from morphological principal component analysis. Key for morphological acronyms in Materials and Methods section.

	PC1	PC2	PC3	PC4	PC5	PC6	PC7	PC8	PC9	PC10
**Standard deviation**	1.6979	1.3632	1.1140	1.0262	0.9245	0.7901	0.6980	0.6484	0.6108	0.4532
**Proportion of Variance**	0.2883	0.1858	0.1241	0.1053	0.0855	0.0624	0.0487	0.0420	0.0373	0.0205
**Cumulative Proportion**	0.2883	0.4741	0.5982	0.7035	0.7890	0.8514	0.9001	0.9422	0.9795	1.0000
**eigen**	2.8827	1.8582	1.2411	1.0530	0.8547	0.6242	0.4872	0.4204	0.3730	0.2054
**LTL**	0.1415	-0.4478	0.5027	-0.1039	0.1112	-0.2044	0.4744	-0.4669	0.1291	0.0450
**RTL**	0.2367	-0.3300	0.5369	-0.0694	0.2063	0.1614	-0.5698	0.3848	0.0184	-0.0161
**MS**	0.0128	0.1468	-0.1261	-0.8743	0.3169	0.2255	-0.0245	-0.1637	-0.1024	-0.0941
**DS**	-0.0936	-0.3966	-0.1336	-0.3632	-0.6880	-0.3641	-0.2415	0.0026	-0.0966	-0.1030
**HL**	0.3093	0.2330	0.2428	-0.1275	-0.5837	0.5466	0.2581	0.1104	0.1913	0.1414
**HW**	0.3932	0.3473	0.1758	0.1660	-0.1267	-0.0645	-0.3150	-0.5258	-0.3967	-0.3363
**LFL**	0.4468	0.2071	-0.1580	-0.0860	0.0642	-0.3928	-0.0915	0.0602	0.7190	-0.1941
**RFL**	0.4851	0.1127	-0.0540	-0.1097	0.0625	-0.3876	0.1953	0.3141	-0.4427	0.5001
**LLL**	0.3044	-0.3685	-0.4523	0.1208	0.0417	0.3078	-0.2746	-0.3950	0.1184	0.4588
**RLL**	0.3703	-0.3813	-0.3210	0.1009	0.0692	0.2158	0.3219	0.2453	-0.2056	-0.5889

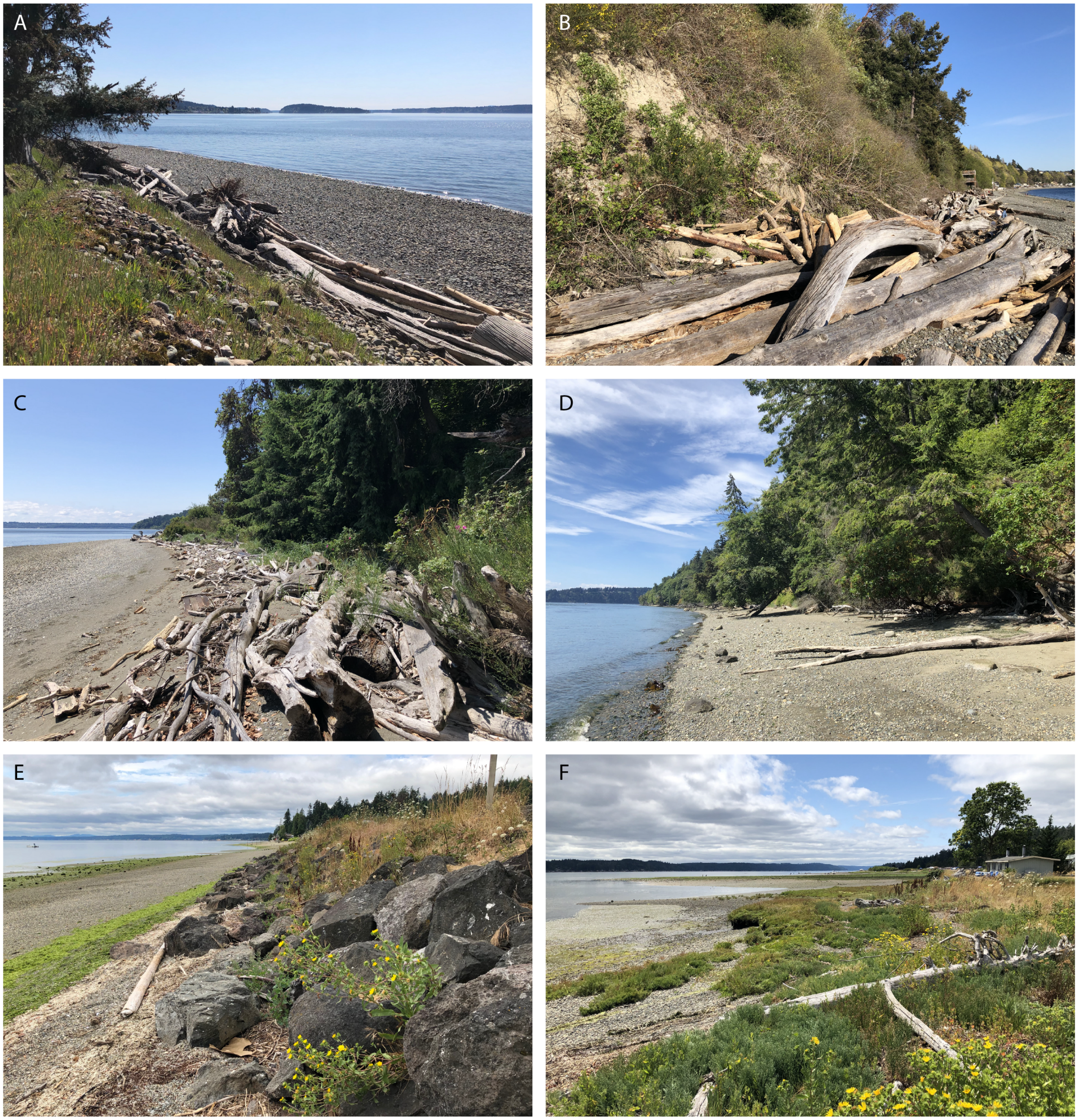

Supplemental Figure 1. Photographs of *Sceloporus occidentalis* habitats in the Puget Sound Region of Western Washington. A) Chambers Creek, near Tacoma, B) Marine View Park, near Burien, C) Maury Island, D) Point Defiance, near Tacoma, E) Purdy Sand Spit, F) Belfair.

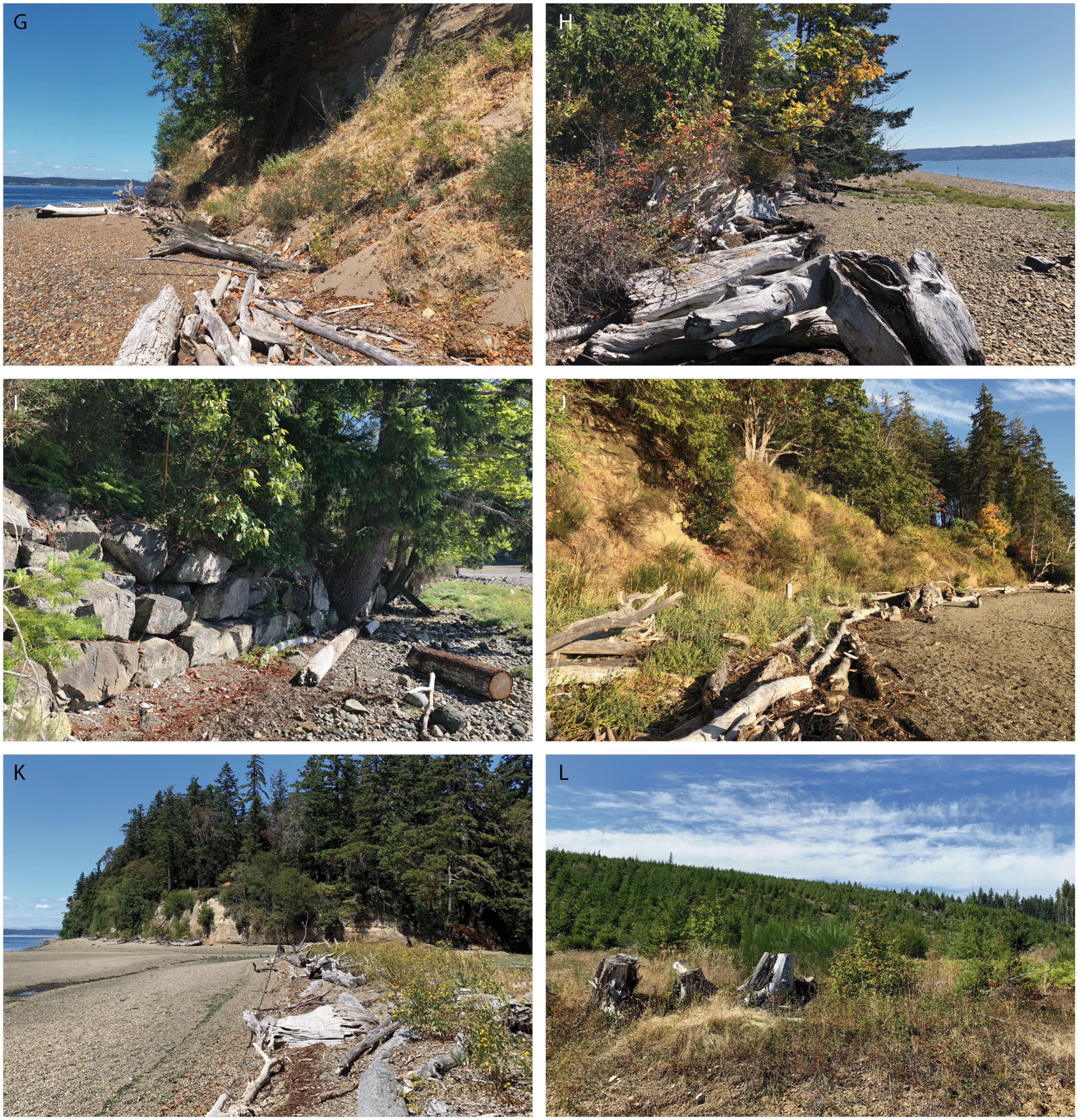

Supplemental Figure 2. Photographs of *Sceloporus occidentalis* habitats in the Puget Sound Region of Western Washington (continued). G) Ketron Island, H) Ayres "Bald" Point, near Tahuya I) Holly, Anderson Cove, J) Joemma Beach, K) Anderson Island, L) Tree Farm, near Dewatto.

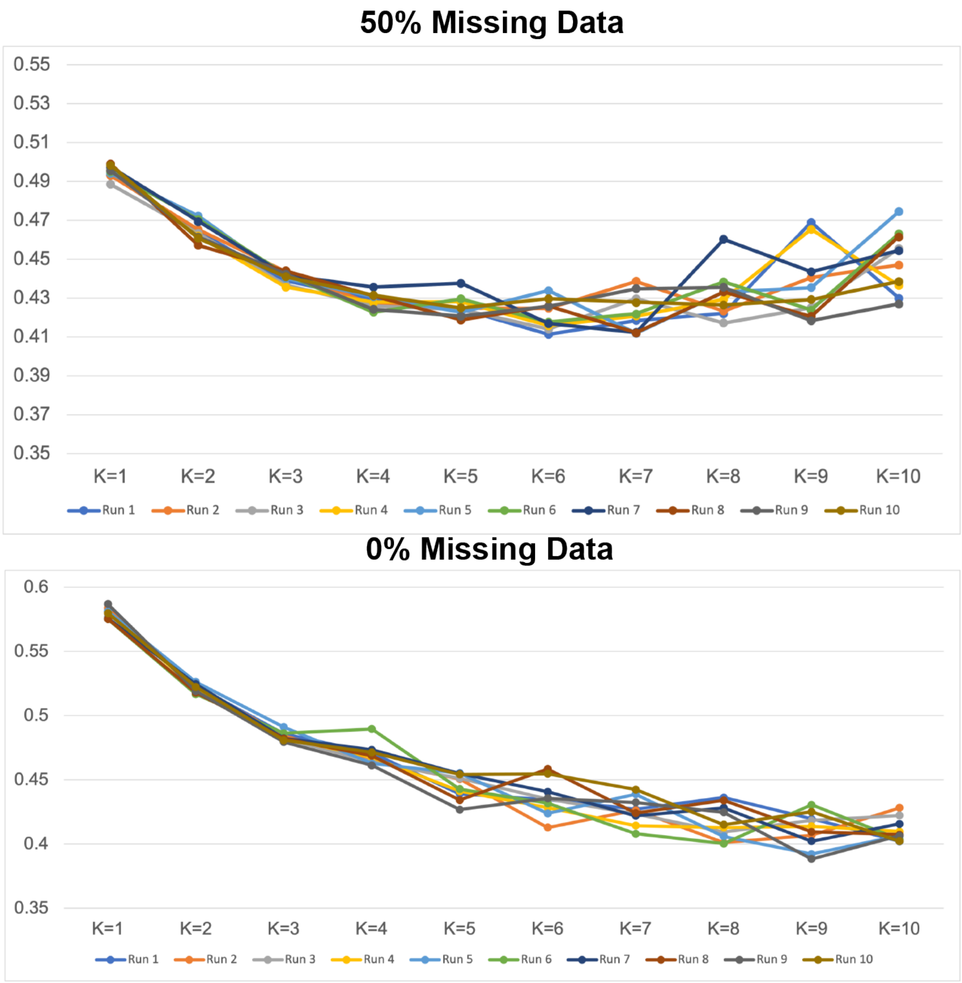

Supplemental Figure 3. Cross-validation scores from the ADMIXTURE analyses for the 50% and 0% missing data datasets, showing that the incorporating more loci lowers the estimated number of subpopulations (*K*). The analyses were repeated 10 times for *K*-values of 1–10, with each colored circle representing an individual run.

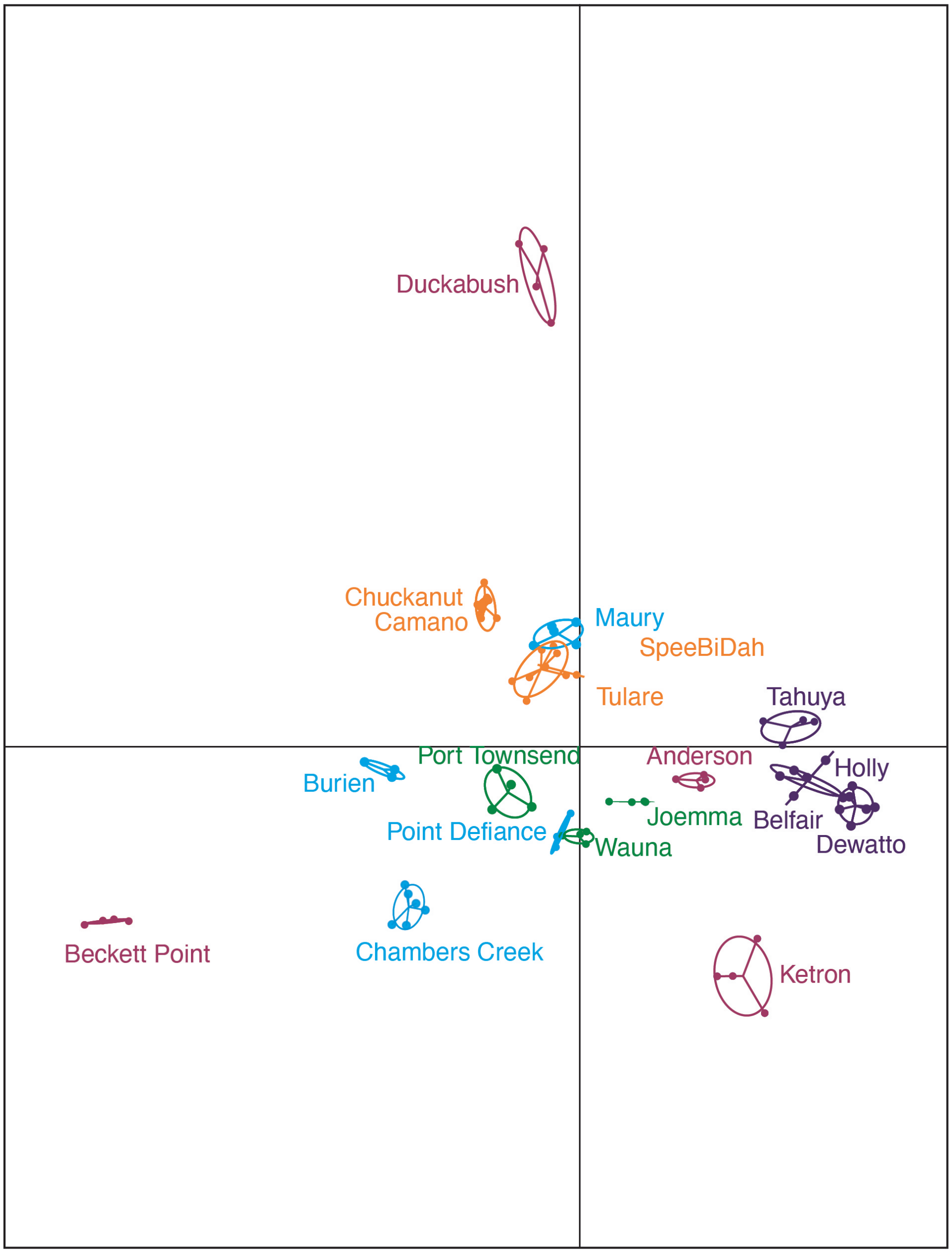

Supplemental Figure 4. Discriminant analysis of principal components of genomic data for *Sceloporus occidentalis* from the Puget Sound region using the R package ’adegenet’.

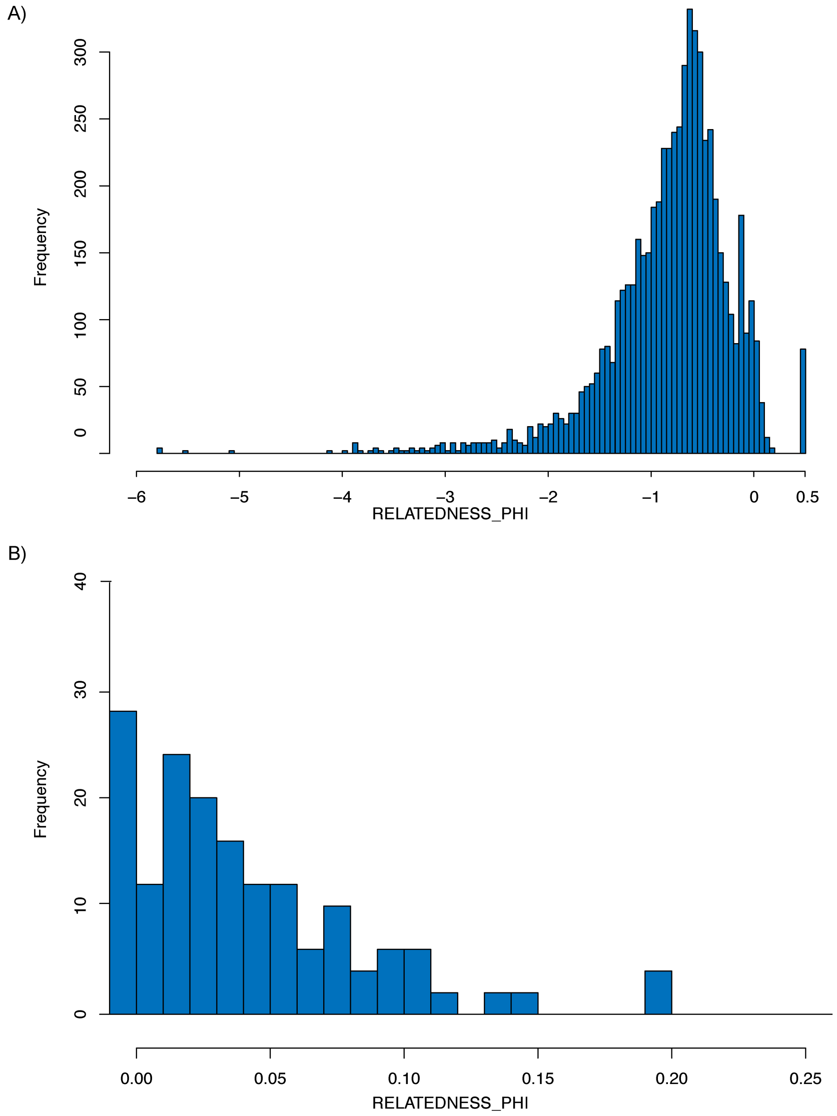

Supplemental Figure 5. Relatedness among samples calculated using the relatedness2 function in VCFtools. Phi is the probability to find identical alleles when randomly sampling one allele from each heterozygous individual. A) Relatedness among all 78 individuals. The majority of comparisons do not include family relationships (phi $\leq$ 0). Phi = 0.5 are self-to-self comparisons. B) Detailed view of relatedness values $\geq$ 0 and < 0.5. There is no evidence for first-degree relatives (phi = 0.25; parents, siblings, offspring). Several comparisons support second degree (phi = 0.125; grandparents, grandchildren, aunts, uncles, nephews, nieces or half-siblings) and third degree (phi = 0.0625; first-cousins, great-grandparents or great-grandchildren) relatives.

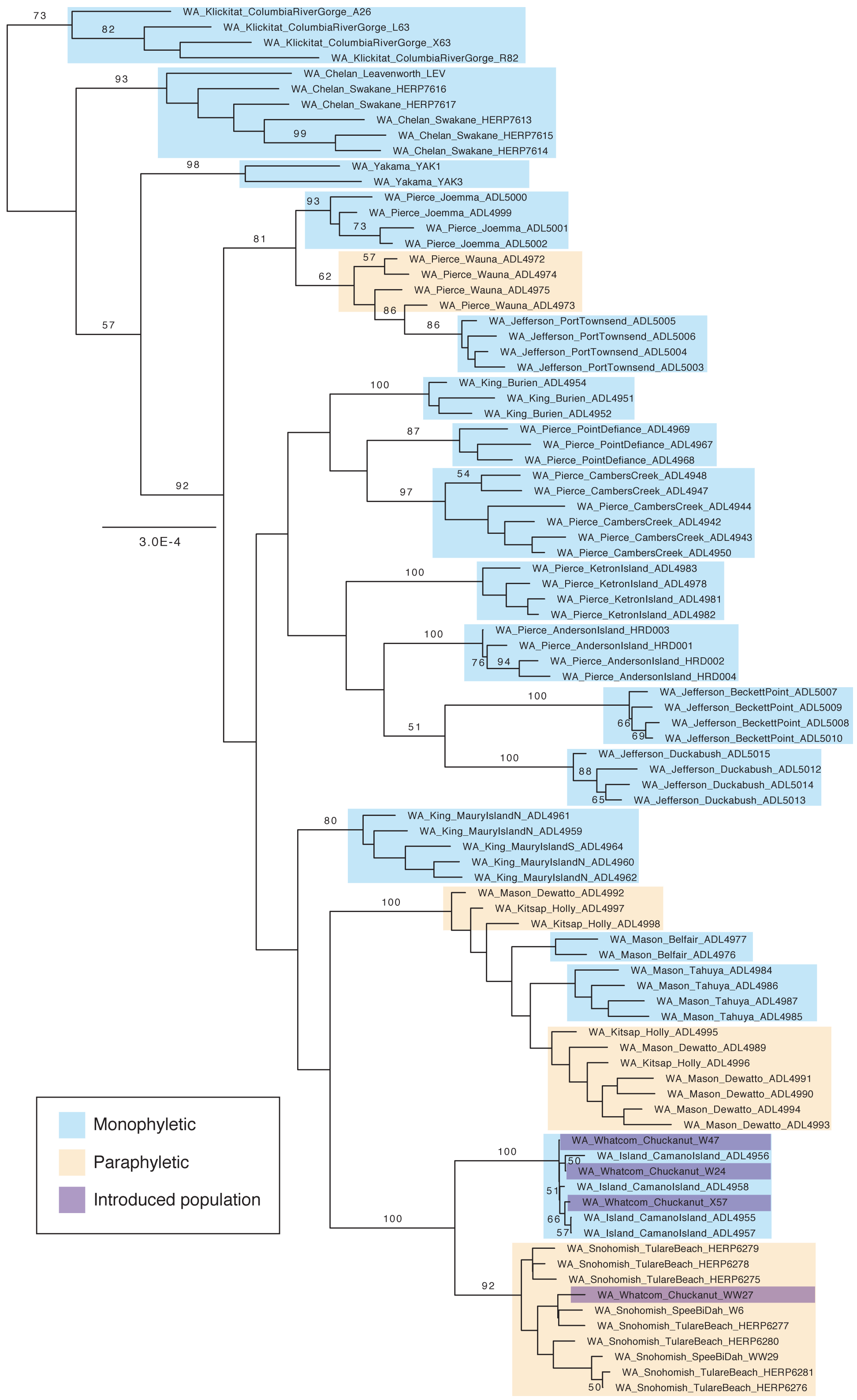

Supplemental Figure 6. Phylogenetic relationships estimated using the concatenated data in RAxML. The tree was rooted using samples from Oregon (not shown). Bootstrap support values ≥ 50% are shown on branches. Color codes indicate if samples collected from a location are monophyletic (blue), paraphyletic (orange), or if they originate from an introduced population (purple).

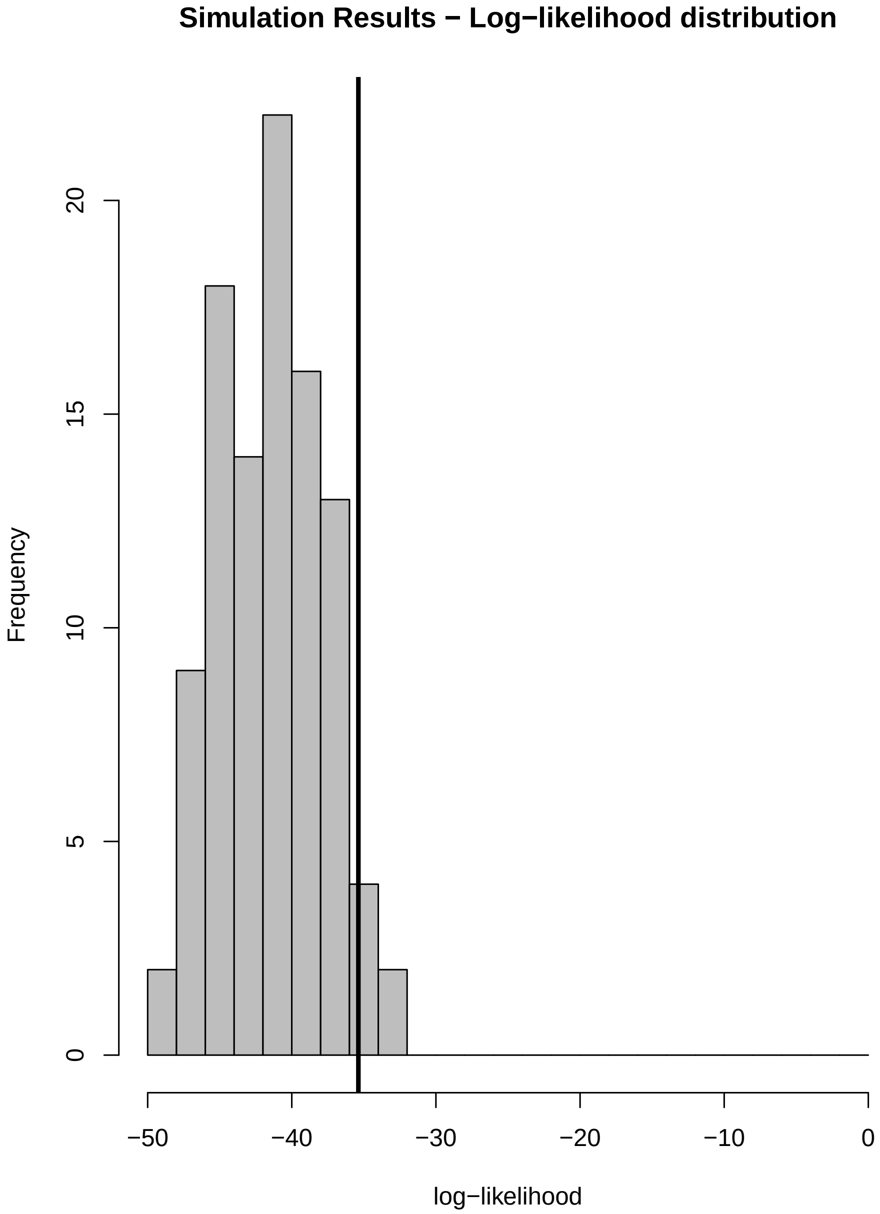

Supplemental Figure 7. Goodness-of-fit test for the demographic models used in the study. The histogram summarizes 100 simulations. The vertical black line shows the results from the empirical data.
